# Adverse stem cell clones within a single patient’s tumor predict clinical outcome in AML patients

**DOI:** 10.1186/s13045-022-01232-4

**Published:** 2022-03-12

**Authors:** Christina Zeller, Daniel Richter, Vindi Jurinovic, Ilse A. Valtierra-Gutiérrez, Ashok Kumar Jayavelu, Matthias Mann, Johannes W. Bagnoli, Ines Hellmann, Tobias Herold, Wolfgang Enard, Binje Vick, Irmela Jeremias

**Affiliations:** 1 Research Unit Apoptosis in Hematopoietic Stem Cells, Helmholtz Zentrum München, German Research Center for Environmental Health (HMGU), Munich, Germany; 2 Anthropology and Human Genomics, Faculty of Biology, Ludwig-Maximilians University (LMU), Munich, Germany; 3 Institute for Medical Information Processing, Biometry and Epidemiology, LMU, Munich, Germany; 4 Department of Proteomics and Signal Transduction, Max Planck Institute of Biochemistry, Martinsried, Germany; 5 Laboratory for Leukemia Diagnostics, Department of Medicine III, University Hospital, LMU, Munich, Germany; 6 German Cancer Consortium (DKTK), Partner Site Munich, Munich, Germany; 7 Department of Pediatrics, Dr. von Hauner Children´s Hospital, University Hospital, LMU, Munich, Germany

**Keywords:** Single cell, Heterogeneity, Xenograft mouse model, Genetic barcoding, In vivo treatment, Therapy resistance

## Abstract

Acute myeloid leukemia (AML) patients suffer dismal prognosis upon treatment resistance. To study functional heterogeneity of resistance, we generated serially transplantable patient-derived xenograft (PDX) models from one patient with AML and twelve clones thereof, each derived from a single stem cell, as proven by genetic barcoding. Transcriptome and exome sequencing segregated clones according to their origin from relapse one or two. Undetectable for sequencing, multiplex fluorochrome-guided competitive in vivo treatment trials identified a subset of relapse two clones as uniquely resistant to cytarabine treatment. Transcriptional and proteomic profiles obtained from resistant PDX clones and refractory AML patients defined a 16-gene score that was predictive of clinical outcome in a large independent patient cohort. Thus, we identified novel genes related to cytarabine resistance and provide proof of concept that intra-tumor heterogeneity reflects inter-tumor heterogeneity in AML.

**Supplementary Information:**

The online version contains supplementary material available at 10.1186/s13045-022-01232-4.

**To the editor**

Acute myeloid leukemia (AML) is difficult to treat and shows major genetic and functional heterogeneity [1, 2]. To complement single cell sequencing studies [3, 4], we characterized single AML stem cells on an in vivo functional level.

From an exemplary and unique AML patient, two relapses, but not the primary diagnostic sample, allowed establishing serially transplantable PDX models, which fulfilled the complex requirements for the planned molecularly guided, clonally diverse, single cell in vivo studies (REL1 and REL2, Figs. 1A and Additional file 2: Figure S1A, Additional file 9: Table S1). Targeted sequencing of AML-specific mutations revealed shared and individual alterations, reflecting clonal heterogeneity and evolution of highly aggressive clones, according to previously published data (Fig. 1B, Additional file 10: Table S2) [5]. Compared to REL1, REL2 showed increased proliferation rates, increased frequency of leukemia initiating cells (LICs), and increased resistance to cytarabine treatment (Additional file 2: Figure S1B-E) [6].

**Fig. 1:**
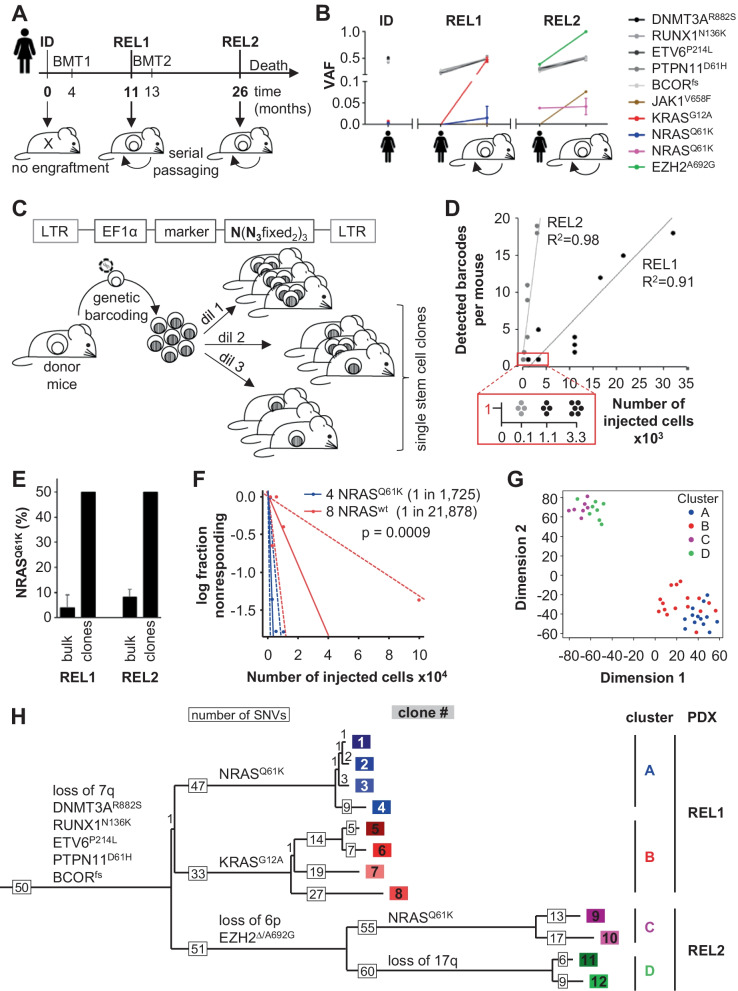
Sequencing divided 12 PDX AML single stem cell clones according to first and second relapse. **A** Primary AML cells from a 52-year-old female patient at time of initial diagnosis (ID), first (REL1) and second relapse (REL2) were transplanted into NSG mice. REL1 and REL2, but not ID, allowed engraftment. **B** Primary tumor (*n* = 1), REL1 PDX (*n* = 9) and REL2 PDX (*n* = 3) cells were analyzed by targeted sequencing. Variant allele frequency (VAF) is depicted. **C–H** Generation and characterization of single PDX AML stem cell clones. **C** Experimental procedure; passage-1 bulk REL1 or REL2 PDX cells were transduced with a genetic barcode and marker^+^ cells injected into mice in limiting dilutions (REL1: 1100–33,000 cells, *n* = 18; REL2: 100–10,000 cells, *n* = 11). At advanced leukemia, PDX cells were re-isolated and barcodes quantified. **D** Numbers of barcodes within REL1 or REL2 populations; one dot represents one mouse. PDX populations consisting of a single barcode were defined as single stem cell clones (red box). **E** NRAS^Q61K^ was determined in PDX clones and compared to proportion of NRAS^Q61K^ cells within bulk REL1 and REL2 PDX cells (mean ± SD, see **B**). **F** Leukemia initiating cell (LIC) frequency of clone 4 (*NRAS*^*Q61K*^) and clone 8 (*NRAS*^*wt*^); cells were injected into mice in limiting dilutions and positive engraftment analyzed. Frequency of LIC and statistical significance was calculated using the ELDA software. Mean (solid line) ± 95% CI (dashed line) is depicted. **G** Gene expression profile was analysed via *prime-seq* from 3–4 biological replicates per clone and a t-distributed stochastic neighbor embedding (t-SNE) plot built by unsupervised clustering. **H** 424 single nucleotide variants (SNVs) were identified from exome sequencing and used to calculate a phylogenetic tree; the length of each branch correlates to number of SNV changes (grey boxes). 50 SNVs of the trunk refer to the complete remission control. Depicted are major chromosomal changes and AML related mutations at each intersection (black), numbers of individual clones (colored boxes), and name of clusters (colored letters)

Aiming for PDX clones originating from a single AML stem cell, we cloned a genetic barcode for first use in PDX models of AML and transplanted cells at limiting dilutions (Fig. 1C, Additional file 1: Supplementary Methods and Additional file 3: Figure S2A) [7, 8]. After growth to end stage leukemia and re-isolation of cells, barcode numbers correlated with numbers of transplanted cells and with LIC frequencies (Fig. 1D and Additional file 3: Figure S2B). In some mice, all PDX cells carried the identical barcode, indicating the engraftment of a single AML stem cell clone; 12 clones allowed reliable serial transplantation, 8 from REL1 and 4 from REL2.

Targeted sequencing revealed that 50% of REL1 and REL2 clones contained the *NRAS*^*Q61K*^ hotspot mutation, although its variant allele frequency was below 10% in both bulk PDX samples (Fig. 1E); accordingly, the LIC frequency of clone 4 (*NRAS*^*Q61K*^*)* was higher compared to clone 8 (*NRAS*^*wt*^*)* (Fig. 1F), indicating an elevated stem cell potential in *NRAS*^*Q61K*^ AML, according to normal hematopoiesis [9].

Transcriptome analysis clustered REL1 apart from REL2 clones (Figs. 1G and Additional file 4: Figure S3AB). Exome sequencing revealed loss of chromosome 7q in REL1 and loss of chromosome 6p in REL2, with clones 11 and 12 showing an additional loss of chromosome 17q (Additional file 4: Figure S3C, Additional file 11: Table S3). Together with 424 single nucleotide variants, exome data inferred a phylogenetic tree which separated REL1 from REL2 and identified 4 clusters (A–D) (Fig. 1H, Additional file 12: Table S4). Taken together, exome and transcriptome mainly divided REL1 from REL2.

The PDX model approach allowed complementing descriptive data with in vivo functional data [8]. In an innovative approach, we marked the clones with individual fluorophore-combinations for flow-cytometric distinction in multiplex competitive in vivo transplantation assays (Figs. 2A and Additional file 5: Figure S4) [10]. REL2 clones harboured slightly elevated homing ability, while REL2 cluster D showed growth advantage over all other clusters (Fig. 2B), with minor inter-mouse variations, indicating biological rather than stochastic effects. Data were reproducible in assays restricted to clones from REL2 with impeded starting conditions for cluster D (Fig. 2C).

**Fig. 2:**
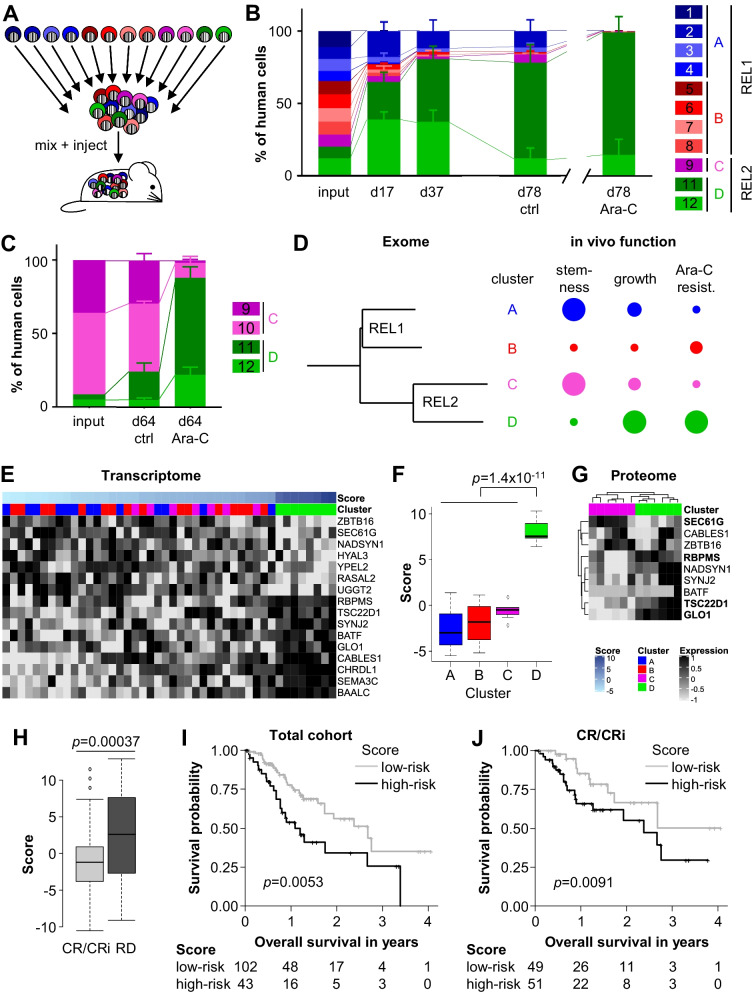
A transcriptome based score from cytarabine resistant PDX clones predicts clinical outcome in AML patients. **A** Experimental procedure; stem cell clones were marked with an individual combination of fluorochromes, mixed and injected into mice for multiplex competitive in vivo experiments. **B** 11 clones were mixed at similar ratios and injected into groups of mice (2 × 10^5^ cells per mouse; *n* = 6 per group). 36d after injection, mice were treated with either PBS (control) or cytarabine (Ara-C). Clonal distribution was determined by flow cytometry at indicated time points. Mean ± SD is depicted. **C** Identical experiment as in **(B)**, except that clones 9–12 were mixed in a 1:1:10:10 ratio (3 × 10^5^ cells per mouse; *n* = 6 per group). Mean ± SD is depicted. **D** Correlation of the phylogenetic tree from Fig. 1H and a summary of the in vivo function; larger circle size indicates increased stemness, faster proliferation or higher Ara-C resistance, respectively. **E** Heatmap showing mRNA expression of the 16 genes of the score in the 12 PDX clones (3–4 biological replicates each, see Supplemental Methods for details on the calculation of the score). Columns were sorted by the score and all variables scaled to the mean value of 0 and variance of 1. **F** The distribution of the predictive score in each cluster; difference between the resistant and the sensitive clusters was calculated with a two-sided *t* test. **G** Heatmap showing protein expression of the 9 genes of the score which were measurable in proteome of REL2 clones (3 biological replicates each); columns were clustered in an unsupervised manner. Proteins with differential expression in the same direction as the corresponding mRNAs are displayed in bold. **H** Association of the predictive score between CR/CRi (*n* = 111) and RD patients (*n* = 46). Two-sided t-test. CR: complete remission; CRi: complete remission with incomplete count recovery; RD: refractory disease. **I, J** Kaplan–Meier plots showing the association between the predictive score and overall survival in the validation cohort (**I**), and in the subcohort of patients who achieved CR/CRi after induction treatment (**J**). The numbers below the x-axis show the patients at risk

Regarding response to chemotherapy, cluster D clones gained clonal dominance upon cytarabine therapy, suggesting increased resistance (Figs. 2BC and Additional file 6: Figure S5). Thus, a discrepancy became visible between sequencing and in vivo functional data; the former separated REL1 from REL2, while the latter identified cluster D as most resistant against cytarabine treatment (Fig. 2D).

To study treatment resistance, we now focused on cluster D which was identified by our unique in vivo functional approach. Transcriptome analysis identified 14 pathways to be enriched, including genes associated with *TGFbeta*, *KRAS* and inflammatory signaling (Additional file 7: Figure S6, Additional file 13: Table S5). In an innovative patient-to-mouse-to-patient approach, we associated genes dysregulated in cluster D with cytarabine resistance in 3 independent cohorts, comprising 1,095 AML patients [11]. A prediction model for cytarabine resistance using penalized logistic regression identified a score of 16 genes that clearly discriminated cluster D from all other clusters (Figs. 2EG, Additional file 1: Supplementary Methods and Additional file 8: Figure S7A). High-resolution mass spectrometry quantified 6894 proteins, with 9/16 score genes present in the proteome, 4 of which showed significant regulation (Fig. 2F). Using an additional independent cohort for validation [12], the 16-gene score was significantly associated with refractory disease, ELN risk groups (Figs. 2H and Additional file 8: S7B), and overall survival (Fig. 2IJ). Moreover, the score was associated with overall and event-free survival in patients with CR/CRi, demonstrating its predictive value beyond induction treatment (Figs. 2IJ and Additional file 8: Figure S7C). Therefore, the score might improve diagnostics of high-risk disease upon putative future routine RNA sequencing.

In summary, our functional in vivo approach on single PDX stem cells linked heterogeneity within a single AML sample to heterogeneity between different samples and provided novel candidate genes associated with cytarabine resistance.

## Supplementary Information

**Additional file 1**. Supplementary Methods.

**Additional file 2**. **Figure S1.** REL2 PDX cells are more resistant towards chemotherapy treatment in vivo than REL1 PDX cells.

**Additional file 3**. **Figure S2.** Quality control of the genetic barcode, related to Fig. 1C, D.

**Additional file 4**. **Figure S3.** Transcriptome analysis and exome sequencing reveal distinct clusters, related to Fig. 1H.

**Additional file 5**. **Figure S4.** Fluorochrome marking of PDX clones enables competitive transplantation experiments, related to Fig. 2.

**Additional file 6**. **Figure S5.** PDX clones display functional differences regarding growth behavior and treatment response in competitive in vivo experiments, related to Fig. 2B, C.

**Additional file 7**. **Figure S6.** Transcriptome analysis reveals enriched pathways in resistant cluster D cells, related to Fig. 2E.

**Additional file 8**. **Figure S7.** AML patients with a high score show poor event-free and overall survival, related to Fig. 2H-J.

**Additional file 9**. **Table S1.** Clinical characteristics of AML patient.

**Additional file 10**. **Table S2.** NGS_panel_seq of patient and bulk PDX samples.

**Additional file 11**. **Table S3.** ExomeCNVs.

**Additional file 12**. **Table S4.** ExomeSNVs.

**Additional file 13**. **Table S5.** Pathway names.

## Data Availability

The gene expression data of the training sets are publicly available through the Gene Expression Omnibus web site (GSE37642, GSE14468, GSE106291). Proteome data are publicly available through the proteome Xchange web site http://www.proteomexchange.org/ Project Name: Adverse stem cell features in a single AML sample predict clinical outcome in AML patient cohorts. Project accession: PXD026296. Project DOI: Not applicable. Reviewer account details: Username: reviewer_pxd026296@ebi.ac.uk. Password: JXueGPuf. NGS, CNV and SNV data can be found in Supplemental tables S2, S3 and S4. For all other original data, please contact binje.vick@hemholtz-muenchen.de.

## Abbreviations

AML: Acute myeloid leukemia
Ara-C: Cytarabine
BMT: Bone marrow transplantation
CI: Confidence interval
CR: Complete remission
CRi: Complete remission with incomplete count recovery
ctrl: Control/solvent treated
d: Day(s)
ELDA: Extreme limiting dilution analysis
ID: Initial diagnosis
LIC: Leukemia initiating cell
NSG: NOD *scid* gamma
PDX: Patient-derived xenograft
RD: Refractory disease
REL: Relapse
SD: Standard deviation
t-SNE: T-distributed stochastic neighbor embedding
VAF: Variant allele frequency
wt: Wildtype
