# Supplementary material for: Adverse stem cell clones within a single patient’s tumor predict clinical outcome in AML patients
Source: J Hematol Oncol. 2022 Mar 12;15:25. doi: 10.1186/s13045-022-01232-4 (PMC8917742; doi:10.1186/s13045-022-01232-4)
Supplement: Supplementary file 1 — Additional file 1. Supplementary Methods. [file 13045_2022_1232_MOESM1_ESM.pdf]

Supplementary Methods

Table of Contents

Ethical Statement 2
The patient-derived xenograft (PDX) model of AML 2
Mouse work 2
Transplantation of primary leukemia cells into NSG mice 3
Monitoring engraftment of PDX cells 3
Experimental end-points 3
Recovering PDX cells from mice and serial transplantation 4
Authentication of passaged PDX cells 4
Enrichment of genetically engineered PDX (GEPDX) cells 4
Bioluminescence *in vivo* imaging (BLI) 5
Quantification of BLI pictures 5
Calculation of doubling time of BLI signals 5
Competitive transplantation assays 5
Flow cytometry analysis of clonal composition 5
In vivo treatment trials 6
Limiting dilution transplantation assay (LDTA) 6
Lentiviral vectors, cloning and transduction 6
Lentiviral vectors 6
Production of lentiviral particles 7
Lentiviral transduction 8
Sequencing 8
Barcode sequencing and data analysis 8
Targeted Sanger sequencing of known subclonal mutations 9
Targeted sequencing of recurrently mutated genes 9
Exome sequencing and data analysis 10
Library preparation and sequencing 10
Single nucleotide variant (SNV) calling 10
Phylogenetic inference 10
Analysis of copy number variants (CNVs) 11
Mass spectrometry (MS) and data analysis 12
Label free proteome sample preparation 12
High pH reversed-phase peptide fractionation 12
Liquid chromatography and mass spectrometry 13
DDA and DIA data analysis 13

Proteome data visualization 14
Data Storage 14
Gene expression profiling, calculation of a predictive score and data analysis 15
Correlation of gene expression of PDX clones to gene expression of AML patients 16
Patient cohorts 16
Statistics 17
Data Sharing Statement 17
References 18

Ethical Statement

Bone marrow (BM) and peripheral blood (PB) samples from an adult acute myeloid leukemia (AML) patient were obtained from the Department of Internal Medicine III, Ludwig-Maximilians-Universität, Munich, Germany. Specimens were collected for diagnostic purposes. Written informed consent was obtained from the patient. The study was performed in accordance with the ethical standards of the responsible committee on human experimentation (written approval by the Research Ethics Boards of the medical faculty of Ludwig-Maximilians-Universität, Munich, number 068-08 and 222-10) and with the Helsinki Declaration of 1975, as revised in 2013.

The patient-derived xenograft (PDX) model of AML

PDX AML samples were established and repetitively passaged as previously described (1, 2).

Mouse work

All animal trials were performed in accordance with the current ethical standards of the official committee on animal experimentation (written approval by Regierung von Oberbayern, tierversuche@reg-ob.bayern.de; ROB-55.2Vet-2532.Vet_02-16-7 and ROB-55.2Vet-2532.Vet_03-16-56). Six to 16 weeks old male and female NOD.Cg-Prkdc^{scid} IL2rg^{tm1Wjl}/SzJ (NSG) mice (The Jackson Laboratory, Bar Harbour, ME, USA) were included. Mice were kept in animal rooms of the Laboratory Animal Breeding and Husbandry Unit of Helmholtz Zentrum München under specified pathogen-free (SPF) conditions with a 12/12 hour light cycle. The animal rooms of the barriers were fully air-conditioned with a temperature of 20-24 °C and 45-65% humidity according to Annex A of the European Convention 2007/526 EC. The maximum stocking density of the

cages corresponds to Annex III of the 2010/63 EU. The cages were constantly filled with structural enrichment and the animals had unlimited access to food and water. During the experiment, mice were kept in individually ventilated cages (IVCs). The cages were only opened one at a time at a cage changing station, the experimenter's gloves were disinfected with disinfectant each time before a mouse was removed from the cage. Hygiene monitoring was carried out at least quarterly in accordance with the current FELASA recommendation: In the animal housing areas equipped with IVC systems, exhaust dust from the IVC ventilation units was tested for all FELASA-listed pathogens by PCR.

Transplantation of primary leukemia cells into NSG mice

Human mononuclear cells were isolated from heparinized BM aspirates or PB by density gradient centrifugation (Ficoll-PaqueTM PLUS, GE Healthcare, Uppsala, Sweden). Cells were washed and suspended in phosphate buffered saline (PBS; Life Technologies, Darmstadt, Germany). 1×10^5 to 1×10^7 fresh or frozen/thawed primary cells were injected into the tail vein of non-irradiated NSG mice. No enrichment or depletion steps were performed.

Monitoring engraftment of PDX cells

To monitor engraftment and growth of human cells in NSG mice, 50 µl of PB was repetitively collected by tail vein aspiration every other week starting from week four after cell injection. Depending on growth kinetics, sampling frequency was increased or decreased. BM was isolated after sacrifice of mice. PB and BM were analyzed by flow cytometry after staining with human CD45 (APC Mouse Anti-Human CD45 Clone HI30, BD Biosciences) and human CD33 (PE Mouse Anti-Human CD33 Clone WM53, BD Biosciences). Antibodies were added to 50 µL of PB or BM cell suspension, and incubated for 30 min. For PB, FACS lysing solution (BD Biosciences, Heidelberg, Germany) was added afterwards and samples were incubated for another 15 min at room temperature. Cells were washed twice with PBS. Flow cytometry was performed with a BD LSRFortessa (BD Biosciences) using CellQuest Pro software (BD). Flow cytometry data was analyzed using FlowJo software (TreeStar Inc., Ashland, OR, USA).

Experimental end-points

The majority of donor mice used for PDX cell amplification were sacrificed at advanced leukemic disease (more than 50% leukemic cells within PB) or when first clinical signs of illness appeared (rough fur, hunchback, reduced motility, paralysis). If 20-30 weeks

after cell injection no clinical signs of illness arose, mice were sacrificed and BM was analyzed by flow cytometry for human cell engraftment. In experimental trials, specific end-points were defined (i.e. x days after injection or treatment). Mice were sacrificed by exposure to CO_2 or cervical dislocation. Mice which died in inhalation narcosis were excluded from further analyses. In therapy trials, mice showing therapy related toxicity (i.e. loss of body weight beyond 15%) were sacrificed and excluded from further analyses. Mice showing leukemia-unrelated illness or peculiarities were sacrificed and excluded from the studies.

Recovering PDX cells from mice and serial transplantation

At advanced leukemic disease, mice were sacrificed and PDX cells were reisolated from bones. Femurs, tibiae, hips, sternum and spine were crushed using mortar and pistil in 10 ml PBS, filtered through a cell strainer, and washed once in PBS. Cells were counted, analyzed by flow cytometry, and viable frozen in 90% FCS / 10% DMSO (5-10x10^6 cells per ml) and stored at -80 °C or liquid nitrogen for long-term storage. Furthermore, 1x10^5 to 5x10^6 PDX cells were reinjected into next recipient NSG mice for expansion or *in vivo* trials. Experimental mice were transplanted either with cells isolated freshly from donor mice, or with frozen/thawed cells from previous donor mice.

Authentication of passaged PDX cells

We worked with our samples PDX AML-491 (REL 1) and AML-661 (REL2). Accuracy of sample identity was regularly verified by repetitive finger printing using PCR of mitochondrial DNA (3).

Enrichment of genetically engineered PDX (GEPDX) cells

To produce GEPDX cells, mice were sacrificed at advanced leukemic disease, and BM cells were isolated from murine BM. GEPDX cells were enriched either by sorting on a FACSAria (BD), or by magnetic cell separation using MicroBeads according to the manufacturer's protocol (Miltenyi Biotech, Bergisch Gladbach, Germany). Enriched GEPDX cells were re-injected into mice for amplification. Enrichment was repeated to achieve >90% transgenic cells. All trials were performed with GEPDX cells purified to levels beyond 90% transgenic.

To analyze GEPDX engraftment at early time points, GEPDX cells were enriched from murine BM via negative selection by MACS using mouse cell depletion kit (Miltenyi) as previously described (1, 4) with the exception that 100-400 µl MicroBeads were used per mouse.

Bioluminescence *in vivo* imaging (BLI)

BLI was performed as previously described (1, 2). Bioluminescence was measured using an IVIS Lumina 2 (Perkin Elmer) to repetitively visualize outgrowth of GEPDX cells in living mice. Mice were transferred to isoflurane inhalation anesthesia. For cells expressing a recombinant codon-optimized form of firefly luciferase, 150 mg/kg D-Luciferin (BIOMOL GmbH, Hamburg, Germany) was injected into the tail vein. Pictures were taken immediately for 30 sec or up to 2 min using a field of view of 12.5 cm with binning 8, f/stop 1 and open filter setting. Pictures with more than 1000 counts were considered within the linear range; saturated pictures were repeated with reduced exposure time or reduced f-stop.

Quantification of BLI pictures

Quantification of light emission was performed using the Living Image software (Caliper Life Sciences, Mainz, Germany). A region of interest (ROI) covering the whole mouse was used and total flux was determined. Values are always depicted in a logarithmic scale.

Calculation of doubling time of BLI signals

To calculate doubling times, quantified BLI signals of untreated mice were collected between 2014 and 2020. For each individual mouse, an exponential trend line was adjusted within the exponential growth phase of PDX cells in mice (total flux between $1x10^7$ and $1x10^{10}$ Photons / second). Doubling time in days was calculated by dividing ln(2)/k, with k being the exponential growth coefficient.

Competitive transplantation assays

PDX clones were thawed, cultured for 4 days in StemPro-34 medium (see above), sorted for fluorochrome expression, mixed in defined ratios and transplanted into groups of recipient mice ($2.6x10^5$ – $6.4x10^5$ cells per mouse). A small portion of the input mix was analyzed by flow cytometry. At defined days post transplantation, PDX cells were reisolated from murine BM and reanalyzed by flow cytometry for proportion of individual clones. Due to technical limitations, clone 7 (mtagBFP+) could not be reliably quantified in murine BM 3 days after injection. Clone 10 had to be excluded in some trials due to insufficient cell numbers.

Flow cytometry analysis of clonal composition

Isolated and enriched BM cells were analyzed using a LSRFortessa X20 (BD Bioscience, Heidelberg, Germany) to determine fluorochrome distributions. Forward/ side scatter analysis was used to gate on living cells, followed by gating on

fluorochromes (mCherry, eGFP, mtagBFP, iRFP720). Data was analyzed using the FlowJo 10 software (FlowJo LLC, Ashley, OR, USA).

In vivo treatment trials

PDX AML bulk cells or (mixed) PDX clones were injected into groups of mice, and tumor burden was regularly monitored by BLI. After reaching an intermediate tumor burden (total flux between $8x10^8$ and $4x10^9$ Photons/second), mice were treated with the following drugs: cytarabine (100 mg/kg dissolved in PBS, i.p.; Cell Pharma GmbH, Bad Vilbel, Germany) was administered on days 2-5 of therapy weeks for up to 4 consecutive weeks or for up to 6 weeks in a bi-weekly rhythm. Weekly cytarabine treatment lead to weight loss of up to 11%, biweekly treatment was well tolerated by NSG mice. DaunoXome (1 mg/kg dissolved in H_2O, i.v.; Galen Ltd, Craigavon, United Kingdom) was administered on days 2 and 5 of therapy weeks. DaunoXome lead to weight loss of up to 8% in NSG mice.

Limiting dilution transplantation assay (LDTA)

For generation of PDX clones, PDX AML cells were isolated from donor mice from passage 1, lentivirally transduced with a genetic barcode library, cultured for 4-6 days in StemPro-34 medium (see above), sorted for marker positive cells and injected into next recipient mice in limiting dilutions. For determination of stem cell frequency of PDX clones 4 and 8, frozen cells were thawed, cultured for 4 days in StemPro-34 medium (see above), counted and injected into next recipient mice in limiting dilutions. Positive engraftment was monitored using BLI or flow cytometric analysis of human AML cells in murine pB. Leukemia initiating cell (LIC) frequency was determined with the ELDA software (http://bioinf.wehi.edu.au/software/elda/) (5).

Lentiviral vectors, cloning and transduction

Lentiviral vectors

Enhanced firefly luciferase vector: https://www.addgene.org/104833/

Barcode vector: For generation of PDX clones, a genetic barcode library was cloned. The barcode vector consisted of a semi-random nucleotide sequence with 10 random nucleotides (N), allowing a theoretical complexity of $4^{10} = 1x10^6$, as well as NGFR for positive selection expressed under the EF1α promoter, flanked by LTR for integration into the host genome. pCDH-EF1α-H2K^k-T2A-NGFR was cloned by replacing the GLuc in pCDH-EF1α-GLuc-T2A-NGFR (Addgene #104832) (4) with a gBlock encoding for H2K^k using the restriction enzymes EcoRI and BamHI. The NheI restriction enzyme

site was destroyed by PCR mutagenesis and, subsequently, an AvrII restriction enzyme site was introduced by cloning of a PCR fragment containing the new restriction enzyme site using KpnI and PciI restriction enzymes. The resulting vector was pre-digested using AvrII and KpnI and dephosphorylated using rSAP. Insert oligos *ExBC forward strand* and *ExBC reverse strand* were annealed to create the barcode-insert by heating equimolar quantities to 90 °C and slow cool down to 8 °C. Barcode-insert cloning into the vector backbone was carried out using a cycling restriction and ligation approach. 100 ng dephosphorylated vector backbone were used with 250 fmol annealed insert, 2.5 U AvrII, 10 U NheI-HF and 400 U T4 DNA Ligase in CutSmart buffer supplemented with 1.5 mM ATP in a total volume of 20 µl per reaction. Reactions were cycled 55x with 5 min at 37 °C and 5 min at 20 °C, followed by a heat-inactivation at 80 °C for 20 min. Reactions were pooled and transformed into NEBstable via heat-shock transformation. 90 ml midi cultures were inoculated with 4 transformations each and incubated over night at 37 °C and 220 rpm shaking. Plasmids were prepared using the PureYield Midi Prep Kit (Promega). The resulting plasmid preparations were pooled to create the final barcode plasmid pool.

Fluorochrome vectors: To facilitate competitive multiplex *in vivo* trials, fluorochromes were chosen according to (6-10). Compared to barcoding, fluorochrome marking is advantageous as it enables re-isolating defined live cells at the end of experiments. The fluorochromes mCherry, eGFP, mtagBFP and iRFP720 were cloned into a pCDH backbone. Additionally, constructs were used that had an enhanced firefly luciferase (eFFly) for BLI coupled via T2A to one of the fluorochromes. All genes were expressed under the elongation factor 1α (EF1α) promotor. pCDH-EF1α-eFFly-T2A-mCherry (Addgene: #104833) (4) and pCDH-EF1α-eFFly-T2A-eGFP (Addgene: #104834) (11) were previously described.

Production of lentiviral particles

1x10^7 HEK293T-cells were seeded in a 75 cm^2 flask in 10 ml DMEM medium (Life Technologies) supplemented with 10% FCS (Biochrom AG, Berlin, Germany) and 1% L-Glutamine (Gibco). Cells should be 80% confluent the next day. One day after seeding, 1 ml DMEM without FCS was mixed with 34 µl PEI (1 mg/ml) and lentiviral packaging plasmids: 2.5 µg of pRSV-Rev (https://www.addgene.org/12253/), 5 µg of pMDLg/pRRE (https://www.addgene.org/12251/), 1.25 µg of pMD2.G (https://www.addgene.org/12259/) and 2.5 µg of lentiviral transfer plasmid (see below). DNA/PEI-mix was incubated for 20 min at room temperature. Meanwhile, medium of

HEK293T-cells was replaced by fresh DMEM + 10% FCS + 1% L-Glutamine. DNA-mix was added dropwise to the cells. Cells were cultivated at 37 °C, 5% CO_2. Three days after transfection, supernatant was transferred into a 15 ml tube and centrifuged for 5 min at 400 g to get rid of residual cells and debris. Supernatant was filtered through a 0.45 µm filter and afterwards transferred to an Amicon Ultra-15 tube (Millipore). Centrifugation for 30 min at 2,000 g resulted in around 200 µl of virus concentrate. This was stored at -80 °C for up to 2 years in 10 µl aliquots.

Lentiviral transduction

At advanced leukemic disease, donor mice were sacrificed, and PDX cells were isolated from murine BM and resuspended in medium. $5x10^6$ cells in 1 ml medium were transferred into a 6 well cell culture plate and transduced overnight with lentiviral constructs in the presence of 8 µg/ml polybrene (Sigma-Aldrich). Transductions were performed at low MOI to guarantee single integrations. After 24 h, cells were washed three times with sterile filtered PBS and re-suspended in medium. After 5-7 days, marker positive cells were sorted and injected into recipient mice for amplification.

AML PDX cells were cultivated in StemPro-34 medium (Thermo Fisher Scientific Waltham, MA, USA) supplemented with 1% L-Glutamin, 1% Penicillin/Streptomycin, 2% FCS (all Gibco, San Diego, CA, USA), 10 ng/ml FLT3L (R&D Systems, Minneapolis, MN, USA), 10 ng/ml SCF, 10 ng/ml TPO, and 10 ng/ml IL3 (all Peprotech, Rocky Hill, NJ, USA) as proposed recently (12) at a density of $1x10^6$ cells per ml, and kept at 37 °C, 5% CO_2.

NALM-6 cells (DSMZ, Braunschweig, Germany) were cultivated in RPMI medium supplemented with 1% L-Glutamin, 10% FCS (all Gibco) and kept at 37 °C, 5% CO_2. Cells were tested on a regular basis for mycoplasma contamination using the MycoAlertTM Mycoplasma Detection Kit (Lonza, Köln, Germany).

Sequencing

Barcode sequencing and data analysis

Genetic barcoding was performed according to (13-18), and their use in PDX models of ALL (19-22) and murine models of AML (23-26).

gDNA was prepared using the Blood&Tissue Kit (QIAGEN) for samples with $\geq 1x10^6$ cells or using QIAamp DNA Micro Kit (QIAGEN) for samples with less than $1x10^6$ cells. For PCR amplification of barcodes a modified SiMSen-Seq (27) approach was used.

For each sample, library preparations were carried out in technical triplicates. In short, 3-5 µl of gDNA were used as input for a first PCR reaction using Phusion II HotStart polymerase (Thermo Fisher Scientific) including primers targeting barcodes and carrying unique molecular identifiers (ExBC_AmpSeq fwd & ExBC_AmpSeq rev; Table S6). After 2-12 cycles, depending on the amount of input gDNA, the reaction was inactivated by addition of 20 µl Actinase E protease (45 ng/µl in buffer TE) and incubated for 15 min at 65 °C, followed by heat-inactivation at 95 °C for 15 min. Subsequently, for each technical replicate of the first PCR two downstream PCRs, each in 40 µl, were carried out using Q5 HotStart High Fidelity Polymerase, 10 µl of the previous reaction as template and 400 nM primers carrying indexed Illumina adapter sequences (Illumina P5 primer & Illumina P7 primer; Table S6). After 20-33 cycles of the second PCR, depending on the initial gDNA input and on-target ratio, samples were cleaned up using SPRI beads at a ratio of 0.8:1 and reactions of the same technical replicates were pooled when eluting with 20 µl buffer EB (Qiagen). Prepared libraries were quantified using a Quant-iT PicoGreen dsDNA assay (Thermo Fisher Scientific) and pooled based on their concentration. Library pools were sequenced using 100 nt single-end dual-indexing runs utiizing a HiSeq1500 (Illumina). Raw fastq files were demultiplexed using deML (28). Specific barcode sequences were extracted from the reads using bartender (29). Barcodes were clustered based on their Hamming distance either using bartender (29) or Starcode (30). Hamming distance thresholds for clustering were set to 3.

Targeted Sanger sequencing of known subclonal mutations

Primers were designed to amplify mutations in *KRAS*, *NRAS* and *EZH2* of the patient detected by targeted sequencing (*KRAS*: fwd: GGCCTGCTGAAAATGACTGA, rev: TGTATCAAAGAATGGTCCTGCA; *NRAS*: fwd: TTGGTCTCTCATGGCACTGT, rev: AGCATTGCATTCCCTGTGGT; *EZH2*: fwd: GCCCTTAGAGATCATGCTAG, rev: CCTGGAACAATAGTGTGTTC). Mutated regions were amplified from isolated gDNA from PDX clones and Sanger sequenced allowing categorization of mutations into mutational frequencies of 0%, 50%, or 100% using the Geneious 11 software (Biomatters Ltd, Auckland, New Zealand).

Targeted sequencing of recurrently mutated genes

68 genes recurrently mutated in myeloid malignancies were sequenced using a targeted amplicon-based enrichment assay (Haloplex, Agilent, Boeblingen, Germany) as previously described (31). Patient material was analyzed once; PDX AML cells were

analyzed in biological replicates: REL1 n=9 (1st to 7th engraftment round), REL2 n=3 (1st and 2nd engraftment round). The variants *DNMT3A*^{R882S}, *RUNX1*^{N136K}, *ETV6*^{P214L}, *PTPN11*^{D61H} and *BCOR*^{P683fs} were present in all samples at variant allele frequencies of approximately 50%, indicating heterozygous mutations in the founding clone. Furthermore, a heterozygous *EZH2*^{A692G} mutation emerged in the second relapse. Additional minor clones appeared with mutations in *KRAS*^{G12A}, *NRAS*^{Q61K} or *JAK1*^{V658F}.

Exome sequencing and data analysis

Library preparation and sequencing: Primary patient material (initial diagnosis, first relapse, and second relapse), controls (first remission, remission after first BM transplant, remission after second BM transplant) and PDX clones were prepared for exome sequencing using SureSelect Human All Exone V6 kit (Agilent Technologies, Santa Clara, CA, USA). Libraries were 100 bp paired-end sequenced using a HiSeq 1500 sequencer (Illumina, San Diego, CA, USA).

Single nucleotide variant (SNV) calling: Paired-end 100 bp whole exome sequencing (WES) reads were mapped using BWA-MEM version 0.7.15-r1140 (32). Reads from patient samples were mapped to the human reference genome, version hg19. Reads from the 12 PDX clones were mapped to a concatenated human-mouse reference genome (hs37d5 - GRCm38), and reads mapping to standard human chromosomes were extracted with samtools version 1.8 (33). SNVs and indels were called following the GATK best practices with MuTect2, version 3.6 (34, 35), using the complete remission and BM donor samples as normal controls. Single-nucleotide polymorphisms (SNPs) and germline indels in both the clones and original patient samples were called with GATK HaplotypeCaller v3.6 (36). The ExAC release 0.3.1 was used as a polymorphism reference (37). We filtered the SNPs per sample according to the GATK best practices by removing SNPs with a quality depth < 2, a Fisher strand test > 60, mean quality < 40, MQRankSum < -12.5, and ReadPosRankSum < -8.0. Indels were filtered with quality depth < 2, a Fisher strand test > 60, and ReadPosRankSum < -20.0. A core set of heterozygous SNPs across samples was defined by the intersection of all SNPs and indels that passed the filters in the diagnosis and full remission samples, were annotated in ExAC, and had a minimum coverage of 20 reads.

Phylogenetic inference: We extracted the allele counts of SNVs that passed the default MuTect2 filters in all the PDX clones using the Rsamtools package, version

2.2.1 (38). Since PDX clones originated from a single cell, we expected the somatic variants of each sample to be fixed. We made a binary matrix of SNV detection per sample, which was set to 1 if the SNV had a variant allele frequency > 0.3 in a PDX clone or > 0.15 in a patient sample. We used hierarchical clustering (complete) to group the PDX clones, using the Jaccard (binary) distance. The clustering dendrogram was converted into a phylogenetic tree using the ape package (39).

Analysis of copy number variants (CNVs): Allele-specific CNVs relative to patient samples in complete remission, representing the reference allele, were estimated on the exome data using the MARATHON pipeline (40), which makes use of the CODEX2 and FALCON-X packages (41, 42). It is based on concerted shifts in the observed frequency of heterozygous site. Thus, a relative allelic Copy Number (CN) is estimated per reference allele per sample. If the genotype is a regular diploid, both alleles have a CN = 1. The core set of heterozygous SNPs from the diagnosis and remission exomes was given as input to CODEX2 to normalize the coverage on the patient and PDX tumor samples with respect to the three control samples (first full remission, full remission after first BM transplant, full remission after second BM transplant). FALCON-X was used to call chromosomal regions with allele-specific copy number differences between each tumor-control pair based on the coverage at heterozygous SNPs which were observed in both samples. Copy number estimates were homogenized along stretches of 1 Mbp, and segments were delimited based on a ΔCN = 0.3. This yielded copy number values for the major and minor copy per tumor-control pair. CNV calls were further processed to yield a unique set that was well supported by all controls, with a homogeneous copy number annotation along the largest possible segments. In order to do this, first we obtained the intersection of CNV regions per sample that were called against each control. Afterwards, we merged the intersected CNV regions of all samples using the reduce() function of the GenomicRanges R package (43), and filtered any segments below 10 Mbp in length. To establish segments within the merged regions that had different copy number values among samples, we extracted all the separate segments with the disjoin() function of GenomicRanges, and merged any adjacent segments that were below 1 Mbp in length. We also merged larger segments to the small ones if they were present in less than 3 samples, or more than 12 (out of 15). This strategy kept regional differences in copy number, while preserving the structure of major CNV events that were shared by multiple samples. The final copy number that was reported per segment corresponds

to the mean between the copy number values per control produced by FALCON-X; this mean copy number was weighted by the length of the original CN reported on the sample, where a CN = 1 was assigned to the genomic ranges that were not annotated in the original FALCON-X output of the sample. We extracted the genes from the final CNV regions based on the GRCh37.75 annotation, importing the corresponding GTF file as a txdb object in R. ENSEMBL gene identifiers were converted to their gene names and Entrez Gene ID with the biomaRt package v2.42.0 (44, 45). Pathway enrichment analysis of the genes in CNV regions was performed with the ReactomePA package (46).

Mass spectrometry (MS) and data analysis

Label free proteome sample preparation

FACS sorted human AML PDX cells were lysed in 1% SDC buffer (1% SDC, 100 mM Tris pH 8.5, 40 mM CAA and 10 mM TCEP), incubated on ice for 20 min, boiled at 95 °C, sonicated for 10 min on a Biorupter plus and heated again for 5 min at 95 °C (47). Proteins in the sample were digested overnight at 37 °C with LysC (1:100 ratio) and Trypsin (1:100 ratio). To the peptides 5x volume Isopropanol / 1% TFA was added and vortexed to stop the digestion. The peptides were de-salted on equilibrated styrenedivinylbenzene-reversed phase sulfonated (SDB-RPS) StageTips, washed once in isopropanol / 1% TFA and twice with 0.2% TFA. Purified peptides were eluted with 60 µl of elution buffer (80%, 1.25% NH_4OH). The dried elutes were resuspended in MS loading buffer (2% ACN, 0.1% TFA) and stored at -20 °C until MS measurement.

High pH reversed-phase peptide fractionation

Fractionation was performed at pH 10 on a nanoflow EASY-nLC 1000 (Thermo Fisher Scientific) using a 30 cm × 250 µm C_{18} reversed-phase column (in house prepared). Peptides (40 µg) were separated at a flow rate of 2 µL min^{-1} on a duration of 96 min with a binary gradient starting from 3% B, which was linearly increased to 30% B within 45 min, 40% B within 12 min, 60% B within 5 min, and to 95% B within 10 min before re-equilibration. Fractions were collected into 8 wells (3 collections) by switching the rotor valve of an automated concatenation system (Spider fractionator, PreOmics) in 4 min intervals (48). Peptide fractions (collected in 0.1% formic acid) were speedvac dried, reconstituted in MS loading buffer and stored at -20 °C until MS measurement.

Liquid chromatography and mass spectrometry

A nanoflow HPLC (EASY-nLC1000, Thermo Fisher Scientific) coupled online to a Q Exactive HF-X Hybrid Quadrupole-Orbitrap Mass Spectrometer (Thermo Fischer Scientific) via a nano electrospray ion source was utilized for the sample analysis. Approximately 500-600 ng of peptides were loaded onto a 50 cm column with 75 µM diameter, packed in house with 1.9 µM C18 ReproSil particles (Dr. Maisch GmbH). The column temperature was maintained using a homemade column oven at 60 °C. The peptides were separated with the binary buffer system of 0.1% formic acid (buffer A) and 60% ACN plus 0.1% formic acid (buffer B), at a flow rate of 300 nl/min. Peptides were eluted on a duration of 120 min with a gradient of 30% buffer B over 95 min and increased to 60% over 5 min. This was followed by a quick ramp up to 95% over 5 min and declined back to 5% over 5 min to re-equilibrate the column.

For experiments involving data-independent data acquisition (DIA), the Thermo Xcalibur (3.0.63) software was used for the Q Exactive HF-X instrument. The Q Exactive HF-X was operated with scan range set to 350-1450 m/z at a resolution of 60,000. The automatic gain control (AGC) was set to $3x10^6$ at a maximum injection time of 54 ms. HCD (NCD 27%) was used for precursor fragmentation and fragment ions were analyzed in 32 DIA windows at a resolution of 30,000, while the AGC was kept at $3x10^6$.

For experiments involving data-dependent data acquisition (DDA), the Thermo Xcalibur (3.0.63) software was used for the Q Exactive HF-X instrument. The Q Exactive HF-X was operated in Top15 mode with a full scan range of 300-1650 m/z at a resolution of 60,000 at 200 m/z. The AGC was set to $3x10^6$ at a maximum injection time of 20 s. Precursor ion selection width was kept at 1.4 m/z and fragmentation was achieved by higher-energy collisional dissociation (HCD) (target $1x10^5$ ions, maximum filling time 120 ms, isolation window 1.6 m/z, and normalized collision energy 27%). Fragment ion scans were recorded at a resolution of 15,000, an AGC of $1x10^5$ and a maximum fill time of 60 ms. Dynamic exclusion was enabled and set to 30 s. Charge exclusion (unassigned, 1,6, -8 & >8) was enabled.

DDA and DIA data analysis

DIA raw files were analyzed using Spectronaut Pulsar (Biognosys, Cat. No. Sw-3001, version 13.12.20021743655) using the default settings for targeted DIA analysis. A project-specific spectral library from PDX samples (8 fractions, DDA data) encompassing 149,249 precursors, corresponding to 8489 protein groups was

generated utilizing pulser against the human Uniprot reference proteome database (version August 2018, containing 21,039 entries) and used. Data export was filtered by ‘No Decoy’ and ‘Quantification Data Filtering’ for peptide and protein quantifications.
In-depth quantitative proteome analysis of REL2 clones was performed using high-resolution mass spectrometry in data-independent mode (DIA). To achieve this in a comprehensive manner, all clones were pooled and separated into 8 fractions by high-pH reverse-phase chromatography. The resulting peptide spectral library contained 8442 proteins obtained in data-dependent mode (DDA). This project-specific proteome library served as a deep spectral library for the sample measured in DIA mode. In total, 6894 proteins were quantified from the 4 clones (average 6800 protein per sample) with high reproducibility between the biological replicates (Pearson correlation, 0.966-0.982) at a protein and peptide false discovery rate of less than 1%. Pairwise comparison of clusters C and D revealed hundreds of proteins differentially regulated between the clones (permutation-based FDR, cutoff 0.05). These proteins were then matched with the transcriptome data to identify the commonly regulated target genes.

Proteome data visualization

We performed data analysis using the open source Perseus environment (version1.5.2.11). DIA analysis achieved data completeness up to 99% per sample. Missing values were imputed by data imputation by normal distribution (Gaussian distribution width=0.5, downshift=1.5) to provide signals for low abundant proteins under the postulation that the low signals is due to detection limit of the MS measurement.

Data Storage

Project Name: Adverse stem cell features in a single AML sample predict clinical outcome in AML patient cohorts

Project accession: PXD026296

Project DOI: Not applicable

Reviewer account details:

Username: reviewer_pxd026296@ebi.ac.uk

Password: JXueGPuf

Differential protein expression was analyzed with the R package *limma* (3.44.3). This data set was used to validated the findings from transcriptome analyses. Genes found to be differentially expressed in transcriptome analysis were considered differentially expressed in the proteome data if their raw p-value was ≤ 0.05.

Gene expression profiling, calculation of a predictive score and data analysis

Gene expression analysis was performed using a modified single cell RNA barcoding and sequencing (Prime-seq) protocol as previously described (1, 49, 50) (see also https://www.protocols.io/view/prime-seq-s9veh66). Libraries were paired-end sequenced on an Illumina HiSeq 1500 instrument (Illumina, San Diego, CA, USA). Sixteen bases were sequenced within the first read to obtain cellular and molecular barcodes, and 50 bases were sequenced in the second read into the cDNA fragment. An additional eight bases were sequenced to obtain the i7 barcode.

All raw fastq data was demultiplexed using deML (28) and further processed with zUMIs (51). Mapping was performed using STAR 2.6.0a (52) against the concatenated human (hg38) and mouse genome (mm10). Gene annotations were obtained from Ensembl (GRCh38.84/GRCm38.85). Samples were identified via the cellular barcode, with initial phred score filtering allowing one base below 20. UMI phred filtering allowed one bases below phred 20.

The raw counts were preprocessed with the R package *edgeR* (3.30.3) and the differential expression between groups was analyzed with the package *limma*. Genes with an adjusted p-value ≤ 0.25 were considered significant.

Within the REL1 and REL2 clusters, we did not observe major differences, including the genes encoded on chromosome 17q that are part of the chromosomal loss in cluster D. Transcriptome analysis identified 993 genes to be differentially expressed in cluster D compared to clusters A-C. For gene set enrichment analysis (GSEA), we analyzed pathways compiled in the Gene Ontology (GO), Kyoto Encyclopedia of Genes and Genomes (KEGG) and Hallmark Gene Sets. After p-value adjustment, 17 pathways were found to be enriched, 14 of which were enriched in the resistant clone; genes associated with *TGFbeta*, *KRAS* and inflammatory signaling tended to be prevalent in cluster D and might contribute to cytarabine resistance.

We compared the list of 993 genes differentially expressed between cluster D and clusters A-C to genes associated with cytarabine resistance in 3 independent cohorts

of AML patients. Gene expression profiles (GEPs) of patients with complete remission (CR) or complete remission with incomplete count recovery (CRi) were compared to the GEPs of patients with refractory disease (RD). Genes that were differentially expressed in the PDX cohort and at least 2 out of 3 patient cohorts were chosen as candidates for a predictive model for cytarabine resistance.

Twenty-three out of 993 genes differentially expressed in cluster D clones showed significant differences in expression levels in at least two patient cohorts. The expression of these 23 genes in the 12 clones was used to construct a prediction model for cytarabine resistance using penalized logistic regression. Seven genes had a regression coefficient of 0 and were thus excluded from the model. The resulting model consisted of 16 genes, with an individual predictive score for each patient calculated as the weighted sum of the 16 gene expression levels multiplied by their model coefficients. A total of 12 out of 16 genes showed differential expression between clusters C and D at a significance value of 0.1, indicating that the difference was not only due to distinct GEPs of REL1 and REL2. As a result, we defined a signature consisting of 16 genes that clearly discriminated cluster D from the remaining clusters A-C.

Correlation of gene expression of PDX clones to gene expression of AML patients

Patient cohorts

Training data set 1 (AMLCG-1) consisted of 407 patients treated within the German AMLCG-1999 trial between 1999 and 2005. This set is a part of a previously published larger data set (GSE37642) that was measured by Affymetrix microarrays (53, 54). Patients were selected if their response to induction treatment was known; patients with a t(15;17) AML or a myelodysplastic syndrome were excluded from the analysis.

Training data set 2 (HOVON) consisted of 449 patients treated in different trials of the Haemato-Oncology Foundation for Adults in the Netherlands (HOVON). This set was also a part of a publicly available cohort analyzed by Affymetrix (GSE14468) (55, 56). Patients were selected based on the availability of information on response to induction treatment.

Training data set 3 (AMLCG-2) consisted of 239 patients treated within the AMLCG-1999 and 2008 trials. Gene expression of this cohort was measured by RNA-Seq (GSE106291).

All 3 training sets have been previously described in (57).

The validation cohort (BEAT) was a subset of 411 patients assessed by the Beat AML program and measured by RNA-Seq (58). We excluded patients younger than 18 years and patients who were not treated with intensive chemotherapy. Relapse samples were also excluded from the analysis. The final validation set consisted of 157 patients.

In order to dichotomize the continuous score in a low- and a high-risk group, we calculated the proportion of evaluable patients who had a refractory disease (37.9%) and used this number as a cutoff for defining the high-risk population (37.9% of patients with the highest risk score).

Statistics

Statistical analyses were calculated using GraphPad Prism 7 software (Graphpad Prism, La Jolla, CA, USA) and R (R Core Team). Repeated measure two-way ANOVA with Sidak correction for multiple testing was applied to test significant differences in BLI curves with and without treatment pressure. Two-tailed t-test with Benjamini and Hochberg correction for multiple testing was applied to test significant differences between calculated PDX clone burden of untreated and treated leukaemias engrafted in mice. Ordinary one-way ANOVA with Tukey correction for multiple testing was used to test significant differences in growth rates between PDX clones as well as PDX clone size after treatment normalized to untreated control. ELDA software was used to determine differences in LIC frequency by Chi-square test ((http://bioinf.wehi.edu.au/software/elda/) (5).

Data Sharing Statement

The gene expression data of the training sets are publicly available through the Gene Expression Omnibus web site (GSE37642, GSE14468, GSE106291).

Proteome data is publicly available through the proteome Xchange web site http://www.proteomexchange.org/

Project Name: Adverse stem cell features in a single AML sample predict clinical outcome in AML patient cohorts

Project accession: PXD026296

Project DOI: Not applicable

Reviewer account details:

Username: reviewer_pxd026296@ebi.ac.uk

Password: JXueGPuf

NGS, CNV and SNV data can be found in Supplemental tables S2, S3 and S4.

For all other original data, please contact binje.vick@hemholtz-muenchen.de

References

1. Ebinger S, Zeller C, Carlet M, Senft D, Bagnoli JW, Liu W-H, et al. Plasticity in growth behavior of patients' acute myeloid leukemia stem cells growing in mice. Haematologica. 2020.

2. Vick B, Rothenberg M, Sandhöfer N, Carlet M, Finkenzeller C, Krupka C, et al. An advanced preclinical mouse model for acute myeloid leukemia using patients' cells of various genetic subgroups and in vivo bioluminescence imaging. PloS one. 2015;10(3):e0120925.

3. Hutter G, Nickenig C, Garritsen H, Hellenkamp F, Hoerning A, Hiddemann W, et al. Use of polymorphisms in the noncoding region of the human mitochondrial genome to identify potential contamination of human leukemia-lymphoma cell lines. The Hematology Journal. 2004;5(1):61-8.

4. Ebinger S, Özdemir EZ, Ziegenhain C, Tiedt S, Alves CC, Grunert M, et al. Characterization of rare, dormant, and therapy-resistant cells in acute lymphoblastic leukemia. Cancer cell. 2016;30(6):849-62.

5. Hu Y, Smyth GK. ELDA: extreme limiting dilution analysis for comparing depleted and enriched populations in stem cell and other assays. Journal of immunological methods. 2009;347(1-2):70-8.

6. Cornils K, Thielecke L, Hüser S, Forgber M, Thomaschewski M, Kleist N, et al. Multiplexing clonality: combining RGB marking and genetic barcoding. Nucleic acids research. 2014;42(7):e56-e.

7. Maetzig T, Morgan M, Schambach A. Fluorescent genetic barcoding for cellular multiplex analyses. Experimental hematology. 2018.

8. Maetzig T, Ruschmann J, Lai CK, Ngom M, Imren S, Rosten P, et al. A lentiviral fluorescent genetic barcoding system for flow cytometry-based multiplex tracking. Molecular Therapy. 2017;25(3):606-20.

9. Weber K, Thomaschewski M, Benten D, Fehse B. RGB marking with lentiviral vectors for multicolor clonal cell tracking. Nature protocols. 2012;7(5):839.

10. Weber K, Thomaschewski M, Warlich M, Volz T, Cornils K, Niebuhr B, et al. RGB marking facilitates multicolor clonal cell tracking. Nat Med. 2011;17(4):504-9.

11. Sandhöfer N, Metzeler K, Rothenberg M, Herold T, Tiedt S, Groiss V, et al. Dual PI3K/mTOR inhibition shows antileukemic activity in MLL-rearranged acute myeloid leukemia. Leukemia. 2015;29(4):828-38.

12. Wermke M, Camgoz A, Paszkowski-Rogacz M, Thieme S, von Bonin M, Dahl A, et al. RNAi profiling of primary human AML cells identifies ROCK1 as a therapeutic target and nominates fasudil as an antileukemic drug. Blood. 2015;125(24):3760-8.

13. Aranyossy T, Thielecke L, Glauche I, Fehse B, Cornils K. Genetic Barcodes Facilitate Competitive Clonal Analyses In Vivo. Hum Gene Ther. 2017;28(10):926-37.

14. Bystrykh LV, Belderbos ME. Clonal analysis of cells with cellular barcoding: when numbers and sizes matter. Stem Cell Heterogeneity: Springer; 2016. p. 57-89.

15. Hyo-eun CB, Ruddy DA, Radhakrishna VK, Caushi JX, Zhao R, Hims MM, et al. Studying clonal dynamics in response to cancer therapy using high-complexity barcoding. Nature medicine. 2015;21(5):440.

16. Kebschull JM, Zador AM. Cellular barcoding: lineage tracing, screening and beyond. Nat Methods. 2018;15(11):871-9.

17. Lu R, Neff NF, Quake SR, Weissman IL. Tracking single hematopoietic stem cells in vivo using high-throughput sequencing in conjunction with viral genetic barcoding. Nature biotechnology. 2011;29(10):928.

18. Schepers K, Swart E, van Heijst JW, Gerlach C, Castrucci M, Sie D, et al. Dissecting T cell lineage relationships by cellular barcoding. Journal of Experimental Medicine. 2008;205(10):2309-18.

19. Belderbos ME, Koster T, Ausema B, Jacobs S, Sowdagar S, Zwart E, et al. Clonal selection and asymmetric distribution of human leukemia in murine xenografts revealed by cellular barcoding. Blood. 2017;129(24):3210-20.

20. Elder A, Bomken S, Wilson I, Blair HJ, Cockell S, Ponthan F, et al. Abundant and equipotent founder cells establish and maintain acute lymphoblastic leukaemia. Leukemia. 2017;31(12):2577.

21. Jacobs S, Ausema A, Zwart E, Weersing E, Kingma MJ, El Menshawi YA, et al. Quantitative distribution of patient-derived leukemia clones in murine xenografts revealed by cellular barcodes. Leukemia. 2019:1-5.

22. Jacobs S, Ausema A, Zwart E, Weersing E, de Haan G, Bystrykh LV, et al. Detection of chemotherapy-resistant patient-derived acute lymphoblastic leukemia clones in murine xenografts using cellular barcodes. Exp Hematol. 2020;91:46-54.

23. Maetzig T, Ruschmann J, Sanchez Milde L, Lai CK, von Krosigk N, Humphries RK. Lentiviral Fluorescent Genetic Barcoding for Multiplex Fate Tracking of Leukemic Cells. Mol Ther Methods Clin Dev. 2017;6:54-65.

24. Oki T, Mercier F, Kato H, Jung Y, McDonald TO, Spencer JA, et al. Imaging dynamic mTORC1 pathway activity in vivo reveals marked shifts that support time-specific inhibitor therapy in AML. Nat Commun. 2021;12(1):245.

25. Chapellier M, Pena-Martinez P, Ramakrishnan R, Eriksson M, Talkhoncheh MS, Orsmark-Pietras C, et al. Arrayed molecular barcoding identifies TNFSF13 as a positive regulator of acute myeloid leukemia-initiating cells. Haematologica. 2019;104(10):2006-16.

26. Klauke K, Broekhuis MJC, Weersing E, Dethmers-Ausema A, Ritsema M, Gonzalez MV, et al. Tracing dynamics and clonal heterogeneity of Cbx7-induced leukemic stem cells by cellular barcoding. Stem Cell Reports. 2015;4(1):74-89.

27. Stahlberg A, Krzyzanowski PM, Egyud M, Filges S, Stein L, Godfrey TE. Simple multiplexed PCR-based barcoding of DNA for ultrasensitive mutation detection by next-generation sequencing. Nat Protoc. 2017;12(4):664-82.

28. Renaud G, Stenzel U, Maricic T, Wiebe V, Kelso J. deML: robust demultiplexing of Illumina sequences using a likelihood-based approach. Bioinformatics. 2015;31(5):770-2.

29. Zhao L, Liu Z, Levy SF, Wu S. Bartender: a fast and accurate clustering algorithm to count barcode reads. Bioinformatics. 2018;34(5):739-47.

30. Zorita E, Cusco P, Filion GJ. Starcode: sequence clustering based on all-pairs search. Bioinformatics. 2015;31(12):1913-9.

31. Metzeler KH, Herold T, Rothenberg-Thurley M, Amler S, Sauerland MC, Görlich D, et al. Spectrum and prognostic relevance of driver gene mutations in acute myeloid leukemia. Blood. 2016;128(5):686-98.

32. Li H. Aligning sequence reads, clone sequences and assembly contigs with BWA-MEM. arXiv preprint arXiv:13033997. 2013.

33. Li H, Handsaker B, Wysoker A, Fennell T, Ruan J, Homer N, et al. The sequence alignment/map format and SAMtools. Bioinformatics. 2009;25(16):2078-9.

34. Cibulskis K, Lawrence MS, Carter SL, Sivachenko A, Jaffe D, Sougnez C, et al. Sensitive detection of somatic point mutations in impure and heterogeneous cancer samples. Nature biotechnology. 2013;31(3):213.

35. Van der Auwera GA, Carneiro MO, Hartl C, Poplin R, Del Angel G, Levy-Moonshine A, et al. From FastQ data to high-confidence variant calls: the genome analysis toolkit best practices pipeline. Current protocols in bioinformatics. 2013;43(1):11-0.

36. Poplin R, Ruano-Rubio V, DePristo MA, Fennell TJ, Carneiro MO, Van der Auwera GA, et al. Scaling accurate genetic variant discovery to tens of thousands of samples. BioRxiv. 2018:201178.

37. Lek M, Karczewski KJ, Minikel EV, Samocha KE, Banks E, Fennell T, et al. Analysis of protein-coding genetic variation in 60,706 humans. Nature. 2016;536(7616):285-91.

38. Morgan M, Pages H, Obenchain V, Hayden N. Rsamtools: Binary Alignment (BAM), FASTA, Variant Call (BCF), and Tabix File Import. 2019.

39. Paradis E, Schliep K. ape 5.0: an environment for modern phylogenetics and evolutionary analyses in R. Bioinformatics. 2019;35(3):526-8.

40. Urrutia E, Chen H, Zhou Z, Zhang NR, Jiang Y. Integrative pipeline for profiling DNA copy number and inferring tumor phylogeny. Bioinformatics. 2018;34(12):2126-8.

41. Chen H, Jiang Y, Maxwell KN, Nathanson KL, Zhang N. Allele-specific copy number estimation by whole exome sequencing. The annals of applied statistics. 2017;11(2):1169.

42. Jiang Y, Wang R, Urrutia E, Anastopoulos IN, Nathanson KL, Zhang NR. CODEX2: full-spectrum copy number variation detection by high-throughput DNA sequencing. Genome biology. 2018;19(1):202.

43. Lawrence M, Huber W, Pages H, Aboyoun P, Carlson M, Gentleman R, et al. Software for computing and annotating genomic ranges. PLoS computational biology. 2013;9(8).

44. Durinck S, Moreau Y, Kasprzyk A, Davis S, De Moor B, Brazma A, et al. BioMart and Bioconductor: a powerful link between biological databases and microarray data analysis. Bioinformatics. 2005;21(16):3439-40.

45. Durinck S, Spellman PT, Birney E, Huber W. Mapping identifiers for the integration of genomic datasets with the R/Bioconductor package biomaRt. Nature protocols. 2009;4(8):1184.

46. Yu G, He Q-Y. ReactomePA: an R/Bioconductor package for reactome pathway analysis and visualization. Molecular BioSystems. 2016;12(2):477-9.

47. Kulak NA, Pichler G, Paron I, Nagaraj N, Mann M. Minimal, encapsulated proteomic-sample processing applied to copy-number estimation in eukaryotic cells. Nat Methods. 2014;11(3):319-24.

48. Kulak NA, Geyer PE, Mann M. Loss-less Nano-fractionator for High Sensitivity, High Coverage Proteomics. Mol Cell Proteomics. 2017;16(4):694-705.

49. Ziegenhain C, Vieth B, Parekh S, Reinius B, Guillaumet-Adkins A, Smets M, et al. Comparative analysis of single-cell RNA sequencing methods. Molecular cell. 2017;65(4):631-43.

50. Janjic A, Wange LE, Bagnoli JW, Geuder J, Nguyen P, Richter D, et al. Prime-seq, efficient and powerful bulk RNA-sequencing. bioRxiv. 2021:2021.09.27.459575.

51. Parekh S, Ziegenhain C, Vieth B, Enard W, Hellmann I. zUMIs - A fast and flexible pipeline to process RNA sequencing data with UMIs. Gigascience. 2018;7(6).

52. Dobin A, Davis CA, Schlesinger F, Drenkow J, Zaleski C, Jha S, et al. STAR: ultrafast universal RNA-seq aligner. Bioinformatics. 2013;29(1):15-21.

53. Herold T, Metzeler KH, Vosberg S, Hartmann L, Rollig C, Stolzel F, et al. Isolated trisomy 13 defines a homogeneous AML subgroup with high frequency of mutations in spliceosome genes and poor prognosis. Blood. 2014;124(8):1304-11.

54. Li Z, Herold T, He C, Valk PJ, Chen P, Jurinovic V, et al. Identification of a 24-gene prognostic signature that improves the European LeukemiaNet risk classification of acute myeloid leukemia: an international collaborative study. J Clin Oncol. 2013;31(9):1172-81.

55. Wouters BJ, Lowenberg B, Erpelinck-Verschueren CA, van Putten WL, Valk PJ, Delwel R. Double CEBPA mutations, but not single CEBPA mutations, define a subgroup of acute myeloid leukemia with a distinctive gene expression profile that is uniquely associated with a favorable outcome. Blood. 2009;113(13):3088-91.

56. Taskesen E, Bullinger L, Corbacioglu A, Sanders MA, Erpelinck CA, Wouters BJ, et al. Prognostic impact, concurrent genetic mutations, and gene expression features of AML with CEBPA mutations in a cohort of 1182 cytogenetically normal AML patients: further evidence for CEBPA double mutant AML as a distinctive disease entity. Blood. 2011;117(8):2469-75.

57. Herold T, Jurinovic V, Batcha AM, Bamopoulos SA, Rothenberg-Thurley M, Ksienzyk B, et al. A 29-gene and cytogenetic score for the prediction of resistance to induction treatment in acute myeloid leukemia. haematologica. 2018;103(3):456-65.

58. Tyner JW, Tognon CE, Bottomly D, Wilmot B, Kurtz SE, Savage SL, et al. Functional genomic landscape of acute myeloid leukaemia. Nature. 2018;562(7728):526-31.
