# Supplementary material for: Adverse stem cell clones within a single patient’s tumor predict clinical outcome in AML patients
Source: J Hematol Oncol. 2022 Mar 12;15:25. doi: 10.1186/s13045-022-01232-4 (PMC8917742; doi:10.1186/s13045-022-01232-4)
Supplement: Supplementary file 2 — Additional file 2. Figure S1. REL2 PDX cells are more resistant towards chemotherapy treatment in vivo than REL1 PDX cells. [file 13045_2022_1232_MOESM2_ESM.pdf]

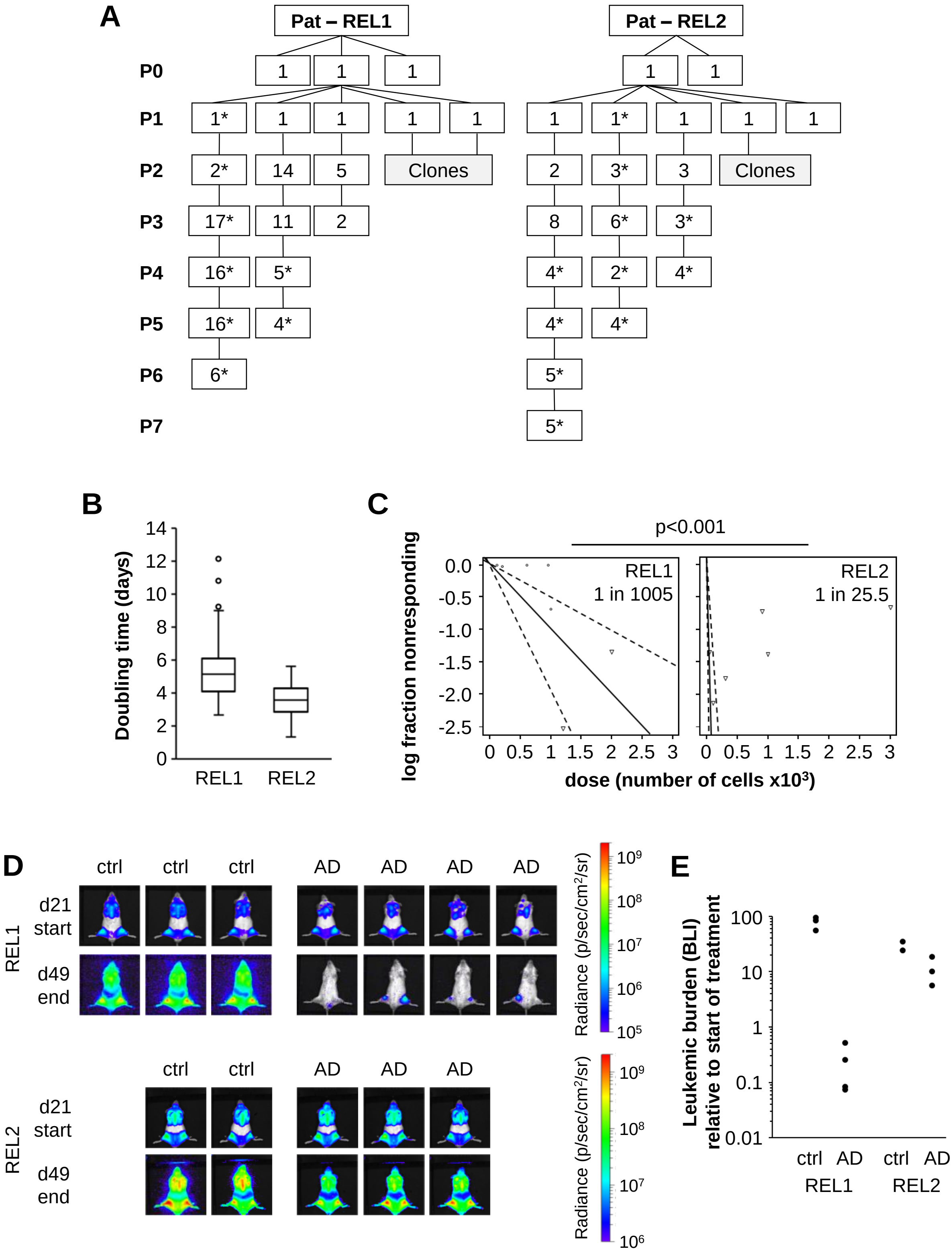
A
Pat – REL1
Pat – REL2
P0
P1
P2
P3
P4
P5
P6
P7
Clones
B
Doubling time (days)
REL1
REL2
C
p<0.001
log fraction nonresponding
REL1
1 in 1005
REL2
1 in 25.5
dose (number of cells x10³)
D
ctrl
AD
d21 start
d49 end
REL1
REL2
Radiance (p/sec/cm²/sr)
E
Leukemic burden (BLI) relative to start of treatment
ctrl
AD
REL1
REL2

Figure S1

Figure S1: REL2 PDX cells are more resistant towards chemotherapy treatment in vivo than REL1 PDX cells.

(A) REL1 and REL2 PDX cells were serially transplanted into NSG mice. Schematic overview of the passaging tree is depicted, with digits indicating the number of mice transplanted in the respective passages (P), and stars (*) indicating genetic engineering of PDX cells by lentiviral transduction. PDX clones were generated from passage 1 cells.

(B) REL1 and REL2 PDX cells were genetically engineered by lentiviral transduction to express enhanced firefly luciferase, sorted for marker$^+$ cells, and amplified. $1x10^5$ to $5x10^6$ cells from different passages were injected into mice (REL1: *n* = 63; REL2: *n* = 49), repeated bioluminescence imaging (BLI) performed and doubling time of BLI signal calculated.

(C) Limiting numbers of luciferase$^+$ REL1 or REL2 PDX cells were transplanted and engraftment monitored by repeated BLI or flow cytometric analysis of PB. Frequency of leukemia initiation cells (LIC) and statistical significance was calculated using the ELDA software. Mean (solid line) ± 95% confidence interval (dashed line) is depicted; $p = 3x10^{-8}$.

(D,E) Luciferase$^+$ REL1 and REL2 PDX cells were injected into mice (*n* = 2 - 4 per group) and tumor burden was regularly monitored by BLI. 21 days (d) after injection, mice were treated with PBS (ctrl) or a combination of Cytarabine and DaunoXome (AD) every second week. Imaging pictures are shown for 21d post injection (start of treatment) and 49d (end of treatment) **(D)**; total flux at 49d relative to 21d is depicted for ctrl- and AD-treated mice **(E)**.
