# Supplementary material for: Adverse stem cell clones within a single patient’s tumor predict clinical outcome in AML patients
Source: J Hematol Oncol. 2022 Mar 12;15:25. doi: 10.1186/s13045-022-01232-4 (PMC8917742; doi:10.1186/s13045-022-01232-4)
Supplement: Supplementary file 3 — Additional file 3. Figure S2. Quality control of the genetic barcode, related to Fig. 1C, D. [file 13045_2022_1232_MOESM3_ESM.pdf]

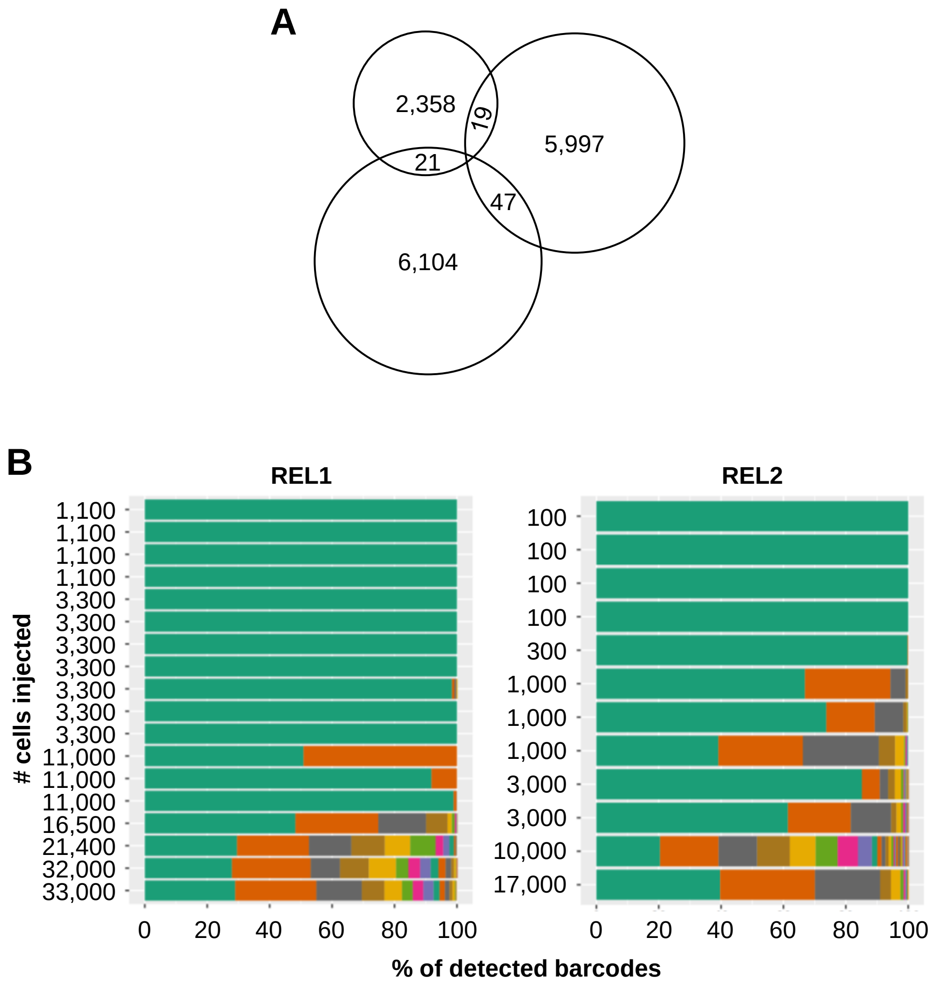

Figure S2. Quality control of the genetic barcode, related to Figures 1C,D.
(A) NALM-6 cells were lentivirally transduced with the low complexity genetic barcode pool in 3 replicates. 4d post transduction, barcodes were amplified from isolated gDNA and sequenced. The Venn-Diagram illustrates number of individual and shared barcodes between replicates. More than 14,000 different barcodes with only minor overlaps between replicates were detected. The complexity of the lentiviral pool was sufficient for generation of single cell clones, as PDX cells were transplanted at low cells numbers, making it unlikely that cells with the same barcode would engraft within one mouse.
(B) Barcode sequencing of exemplary mice; leukemia population isolated from mice engrafted with indicated cell numbers of barcoded PDX AML cells were sequenced for the genetic barcode. Only one color indicates that one single genetic barcode was detectable in a mouse.

Figure S2
