# Supplementary material for: Adverse stem cell clones within a single patient’s tumor predict clinical outcome in AML patients
Source: J Hematol Oncol. 2022 Mar 12;15:25. doi: 10.1186/s13045-022-01232-4 (PMC8917742; doi:10.1186/s13045-022-01232-4)
Supplement: Supplementary file 4 — Additional file 4. Figure S3. Transcriptome analysis and exome sequencing reveal distinct clusters, related to Fig. 1H. [file 13045_2022_1232_MOESM4_ESM.pdf]

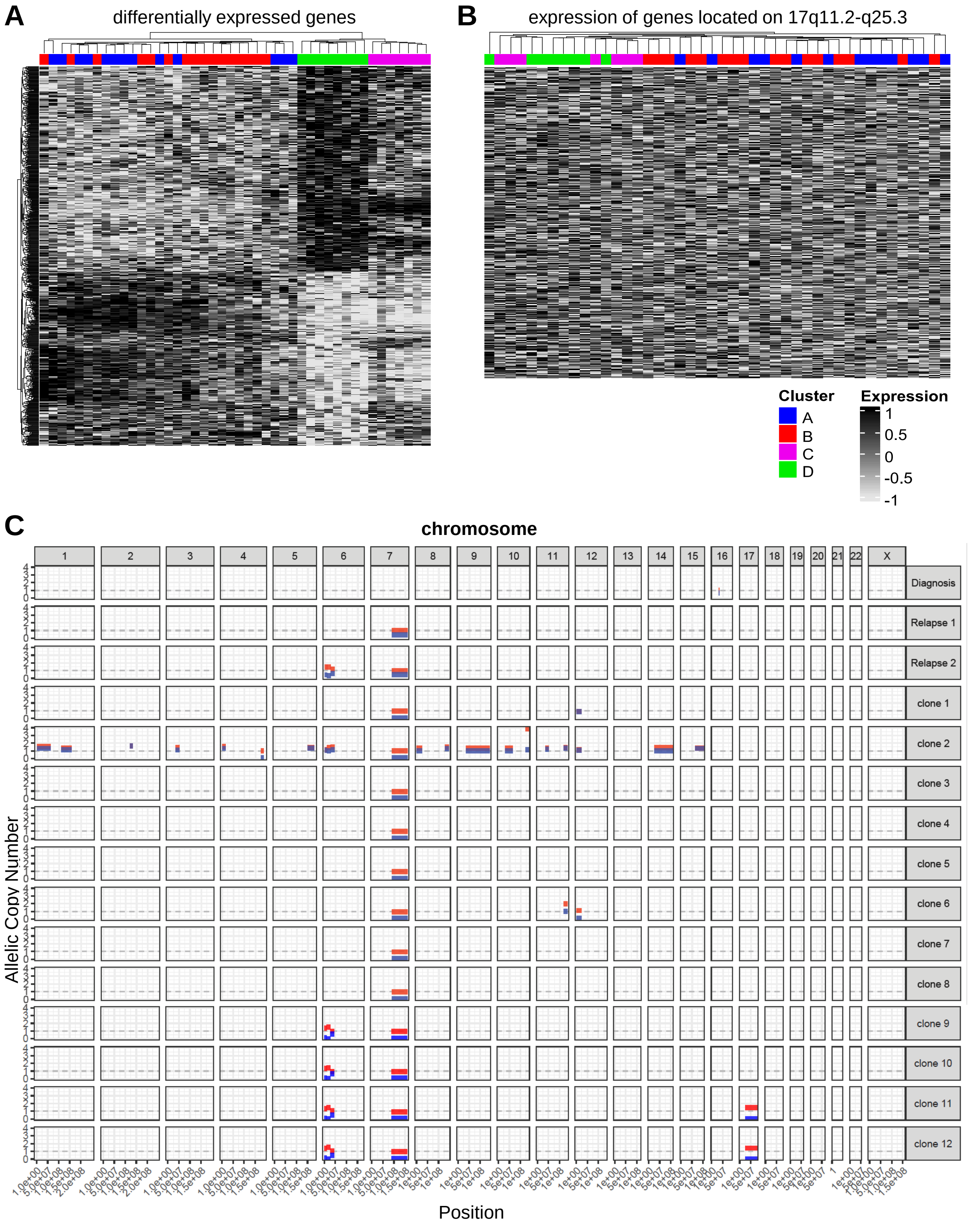
A
differentially expressed genes
B
expression of genes located on 17q11.2-q25.3
Cluster
A
B
C
D
Expression
1
0.5
0
-0.5
-1
C
chromosome
1
2
3
4
5
6
7
8
9
10
11
12
13
14
15
16
17
18
19
20
21
22
X
Diagnosis
Relapse 1
Relapse 2
clone 1
clone 2
clone 3
clone 4
clone 5
clone 6
clone 7
clone 8
clone 9
clone 10
clone 11
clone 12
Allelic Copy Number
Position
allele 1
allele 2
CN = 1

Figure S3

Figure S3. Transcriptome analysis and exome sequencing reveal distinct clusters, related to Figure 1H.

(A,B) RNA was isolated from PDX clones, and transcriptome was analyzed via *prime-seq* from 3 - 4 biological replicates per clone.

(A) Heatmap showing all differentially expressed genes between at least 2 clusters.

(B) Heatmap showing expression of all genes located on 17q11.2-q25.3.

(C) gDNA was isolated from primary tumor cells, PDX clones, and remission samples. Allele specific copy number variations (CNVs) relative to complete remission, representing the reference allele, were estimated using the MARATHON pipeline, which is based on concerted shifts in the observed frequency of heterozygous site. Thus a relative allelic copy number (CN) is estimated per reference allele per sample. A regular diploid genotype is indicated with a CN = 1 (dashed grey line). We detected CNVs with a deviation of at least 10% within 16 chromosomes in different samples (blue and red boxes).
