# Supplementary material for: Adverse stem cell clones within a single patient’s tumor predict clinical outcome in AML patients
Source: J Hematol Oncol. 2022 Mar 12;15:25. doi: 10.1186/s13045-022-01232-4 (PMC8917742; doi:10.1186/s13045-022-01232-4)
Supplement: Supplementary file 5 — Additional file 5. Figure S4. Fluorochrome marking of PDX clones enables competitive transplantation experiments, related to Fig. 2. [file 13045_2022_1232_MOESM5_ESM.pdf]

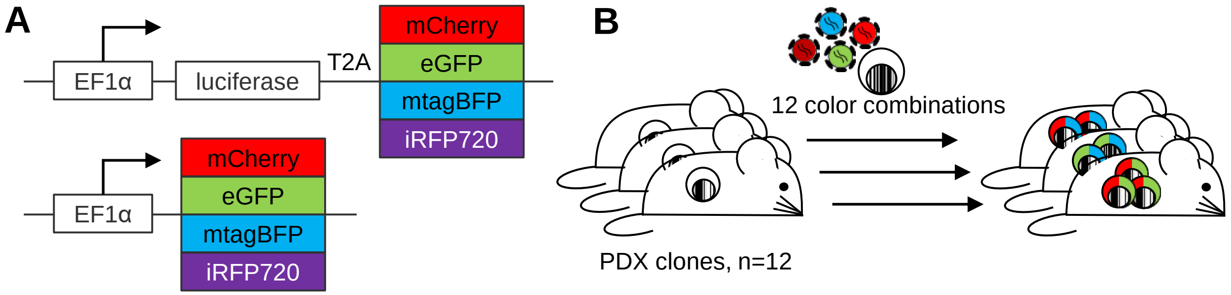

C

clone	sample	cluster	NRAS	KRAS	EZH2	JAK1	color	Fluorochrome combination
1								mCherry+mtagBFP+iRFP
2		A	wt/Q61K	wt/wt	wt/wt	wt/wt		eGFP+mtagBFP+iRFP
3								eGFP
4	REL1							eGFP+iRFP
5								mCherry
6		B	wt/wt	wt/G21A	wt/wt	wt/wt		mCherry+eGFP+mtagBFP
7								mtagBFP
8								iRFP720
9		C	wt/Q61K	wt/wt	Δ/A692G	wt/wt		mtagBFP+iRFP
10	REL2							mCherry+iRFP
11		D	wt/wt	wt/wt	Δ/A692G	wt/wt		eGFP+mtagBFP
12								mCherry+eGFP+mtagBFP+iRFP

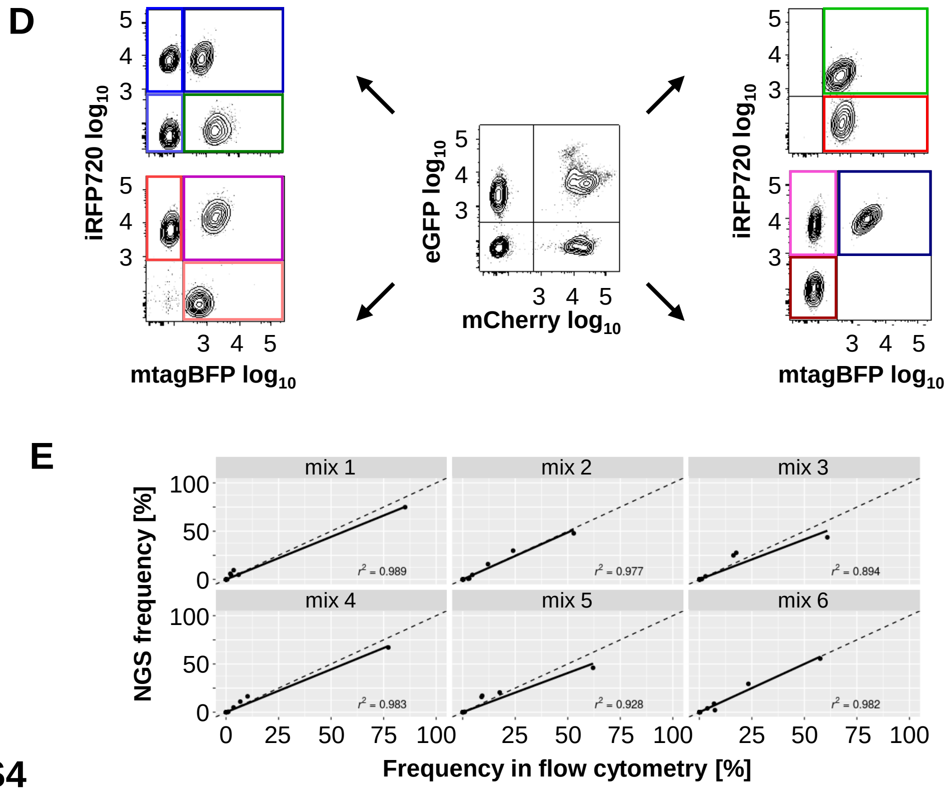

Figure S4

Figure S4. Fluorochrome marking of PDX clones enables competitive transplantation experiments, related to Figure 2.

(A) Scheme of lentiviral constructs used to mark PDX clones with an individual fluorochrome combination of up to 4 fluorochromes and enhanced firefly luciferase to enable BLI. Each of the 4 fluorochromes (mCherry, eGFP, mtagBFP, iRFP720) was expressed with or without enhanced firefly luciferase under the elongation factor 1α (EF1α) promotor.

(B) Experimental procedure; 12 barcode-proven PDX clones were marked with an individual fluorochrome combination and a luciferase by lentiviral transduction. Fluorochrome marked PDX clones were sorted and amplified for competitive functional in vivo analyses.

(C) Table summarizing subclonal mutations, color codes, and fluorochrome combination of established PDX clones.

(D) Schematic flow cytometry gating strategy to discriminate all 12 clones based on individual fluorochrome expressions.

(E) Validation of flow cytometry readout of competitive transplantation experiments compared to barcode readout; fluorochrome marked PDX clones were mixed, injected into mice (n=6), and reanalyzed 78d after injection by flow cytometry and barcode sequencing. Linear regression based on the barcode frequencies from both readouts showed a good agreement between both methods. Therefore FACS analysis was considered an equally suitable but faster method to determine the composition of the mixed populations of single cell clones.
