# Supplementary material for: Adverse stem cell clones within a single patient’s tumor predict clinical outcome in AML patients
Source: J Hematol Oncol. 2022 Mar 12;15:25. doi: 10.1186/s13045-022-01232-4 (PMC8917742; doi:10.1186/s13045-022-01232-4)
Supplement: Supplementary file 6 — Additional file 6. Figure S5. PDX clones display functional differences regarding growth behavior and treatment response in competitive in vivo experiments, related to Fig. 2B, C. [file 13045_2022_1232_MOESM6_ESM.pdf]

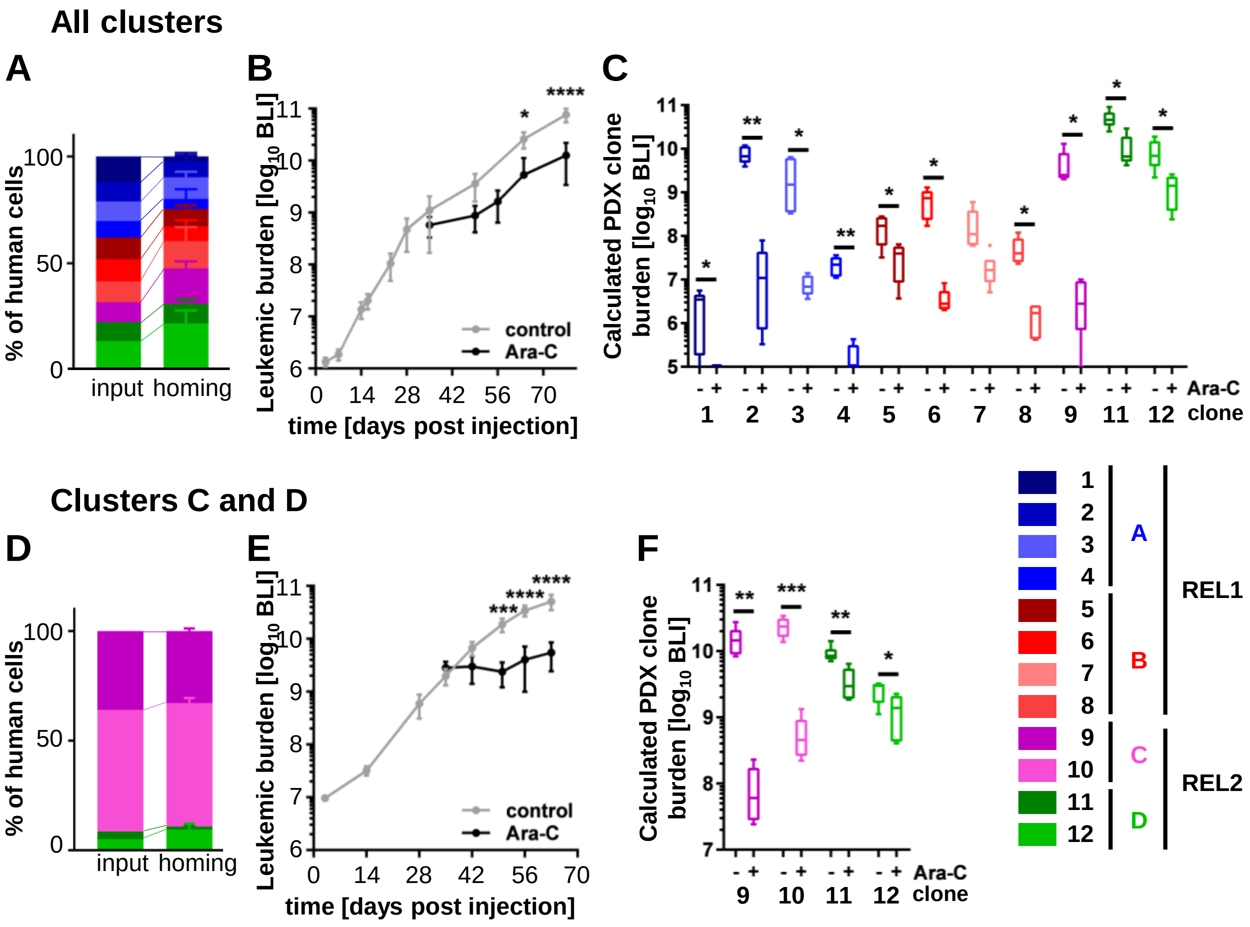
All clusters
A
% of human cells
100
50
0
input
homing
B
Leukemic burden [log10 BLI]
11
10
9
8
7
6
0
14
28
42
56
70
time [days post injection]
*
****
control
Ara-C
C
Calculated PDX clone burden [log10 BLI]
- + Ara-C
1 2 3 4 5 6 7 8 9 11 12 clone
Clusters C and D
D
% of human cells
input
homing
E
Leukemic burden [log10 BLI]
time [days post injection]
***
****
****
control
Ara-C
F
Calculated PDX clone burden [log10 BLI]
- + Ara-C
9 10 11 12 clone
1
2
3
4
5
6
7
8
9
10
11
12
A
B
C
D
REL1
REL2

Figure S5

Figure S5. PDX clones display functional differences regarding growth behavior and treatment response in competitive *in vivo* experiments, related to Figure 3B,C.

(A,B) 11 clones **(A)** or REL2 clones **(B)** were mixed at similar ratios and injected into groups of mice ($2x10^5$ cells per mouse; *n* = 6 per group). 36d after injection, mice were treated with either PBS (control) or cytarabine (Ara-C). Clonal distribution was determined by flow cytometry after homing on day 3 **(C)** and at later indicated time points **(D)**. Mean ± SD is depicted.

(A-C) PDX clones 1-9, 11 and 12 were mixed at a similar ratio and injected into groups of mice ($2x10^5$ cells per mouse; *n* = 6 per group). Due to low cell numbers, clone 10 was excluded from the experiment. Tumor burden was regularly monitored by BLI. At an intermediate tumor burden, mice were either treated with PBS (control) or cytarabine (Ara-C) every second week. **(A)** Clonal distribution was determined by flow cytometry after homing on day 3. **(B)** Quantification of BLI signal; total flux of control (grey) and Ara-C treated mice (black) is depicted (mean ± SD). Statistical significance between control and Ara-C treated mice was tested with two-way ANOVA with Sidak correction. **(C)** 42d after start of therapy, mice were sacrificed and PDX clone composition in murine BM was analyzed by flow cytometry and compared between control and Ara-C treated mice. PDX clone burden was calculated for every mouse using the relative ratio of each individual PDX clone as measured in flow cytometry, multiplied by the BLI quantification of each mouse after end of treatment and is shown as box plots. E.g., a clone covering 5% tumor burden at a BLI of $1x10^9$ P/sec would get a calculated clonal tumor burden of $5x10^7$ P/sec. Statistical significance between control (-) and Ara-C treated mice (+) was tested with t-test with Benjamini and Hochberg correction.

(D-F) PDX clones 9-12 were mixed in a 10:10:1:1 ratio and injected into groups of mice ($3x10^5$ cells per mouse; *n* = 6). Tumor burden was regularly monitored by BLI. At an intermediate tumor burden, mice were either treated with PBS (control) or cytarabine (Ara-C) for 4 consecutive weeks. **(D)** Clonal distribution was determined by flow cytometry after homing on day 3. **(E)** Quantification of BLI signal was performed as described in **(C)**. **(F)** 28d after start of therapy, mice were sacrificed and PDX clone burden was calculated as described in **(D)**.

* $p<0.05$; ** $p<0.01$; *** $p<0.001$; **** $p<0.0001$.
