# Supplementary material for: Adverse stem cell clones within a single patient’s tumor predict clinical outcome in AML patients
Source: J Hematol Oncol. 2022 Mar 12;15:25. doi: 10.1186/s13045-022-01232-4 (PMC8917742; doi:10.1186/s13045-022-01232-4)
Supplement: Supplementary file 7 — Additional file 7. Figure S6. Transcriptome analysis reveals enriched pathways in resistant cluster D cells, related to Fig. 2E. [file 13045_2022_1232_MOESM7_ESM.pdf]

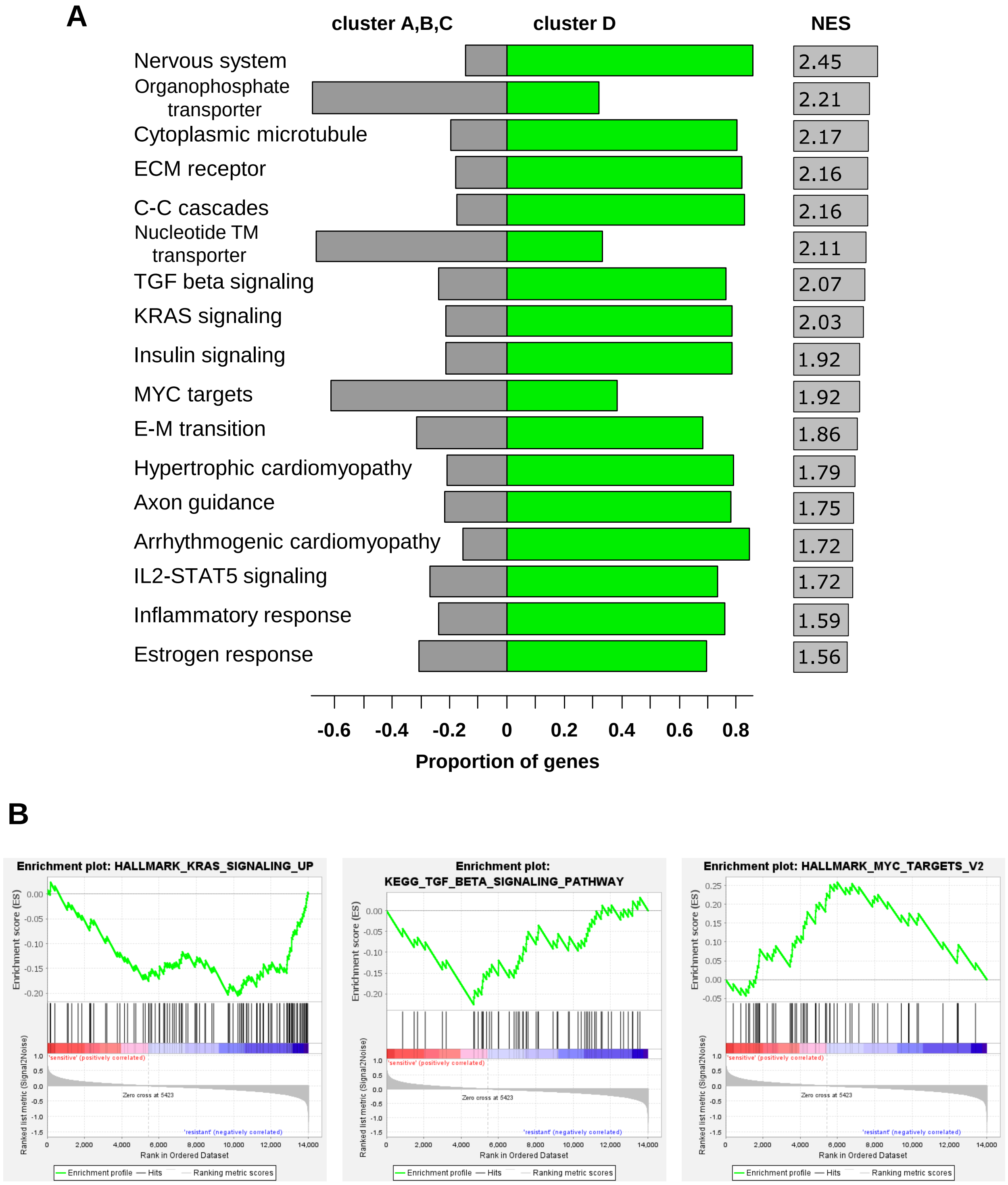
A
cluster A,B,C
cluster D
NES
Nervous system
2.45
Organophosphate transporter
2.21
Cytoplasmic microtubule
2.17
ECM receptor
2.16
C-C cascades
2.16
Nucleotide TM transporter
2.11
TGF beta signaling
2.07
KRAS signaling
2.03
Insulin signaling
1.92
MYC targets
1.92
E-M transition
1.86
Hypertrophic cardiomyopathy
1.79
Axon guidance
1.75
Arrhythmogenic cardiomyopathy
1.72
IL2-STAT5 signaling
1.72
Inflammatory response
1.59
Estrogen response
1.56
-0.6 -0.4 -0.2 0 0.2 0.4 0.6 0.8
Proportion of genes
B
Enrichment plot: HALLMARK_KRAS_SIGNALING_UP
Enrichment plot: KEGG_TGF_BETA_SIGNALING_PATHWAY
Enrichment plot: HALLMARK_MYC_TARGETS_V2
Enrichment score (ES)
Ranked list metric (Signal2Noise)
'sensitive' (positively correlated)
Zero cross at 5423
'resistant' (negatively correlated)
Rank in Ordered Dataset
Enrichment profile
Hits
Ranking metric scores

Figure S6

Figure S6. Transcriptome analysis reveals enriched pathways in resistant cluster D cells, related to Figure 2E.

(A) Gene ontology analysis of differentially expressed genes. Shown are pathways found to be enriched or depleted in resistant clones (green) compared to sensitive clones (grey). All pathways with an adjusted *p*-value ≤ 0.25 were considered significant. The bars indicate the proportion of genes within a pathway that were upregulated in each group. NES: normalized enrichment score. The full pathway names are given in Table S5.

(B) Gene set enrichment analysis (GSEA) of differentially expressed genes within resistant cluster D cells compared to sensitive clusters within 3 pathways.
