# Supplementary material for: Adverse stem cell clones within a single patient’s tumor predict clinical outcome in AML patients
Source: J Hematol Oncol. 2022 Mar 12;15:25. doi: 10.1186/s13045-022-01232-4 (PMC8917742; doi:10.1186/s13045-022-01232-4)
Supplement: Supplementary file 8 — Additional file 8. Figure S7. AML patients with a high score show poor event-free and overall survival, related to Fig. 2H-J. [file 13045_2022_1232_MOESM8_ESM.pdf]

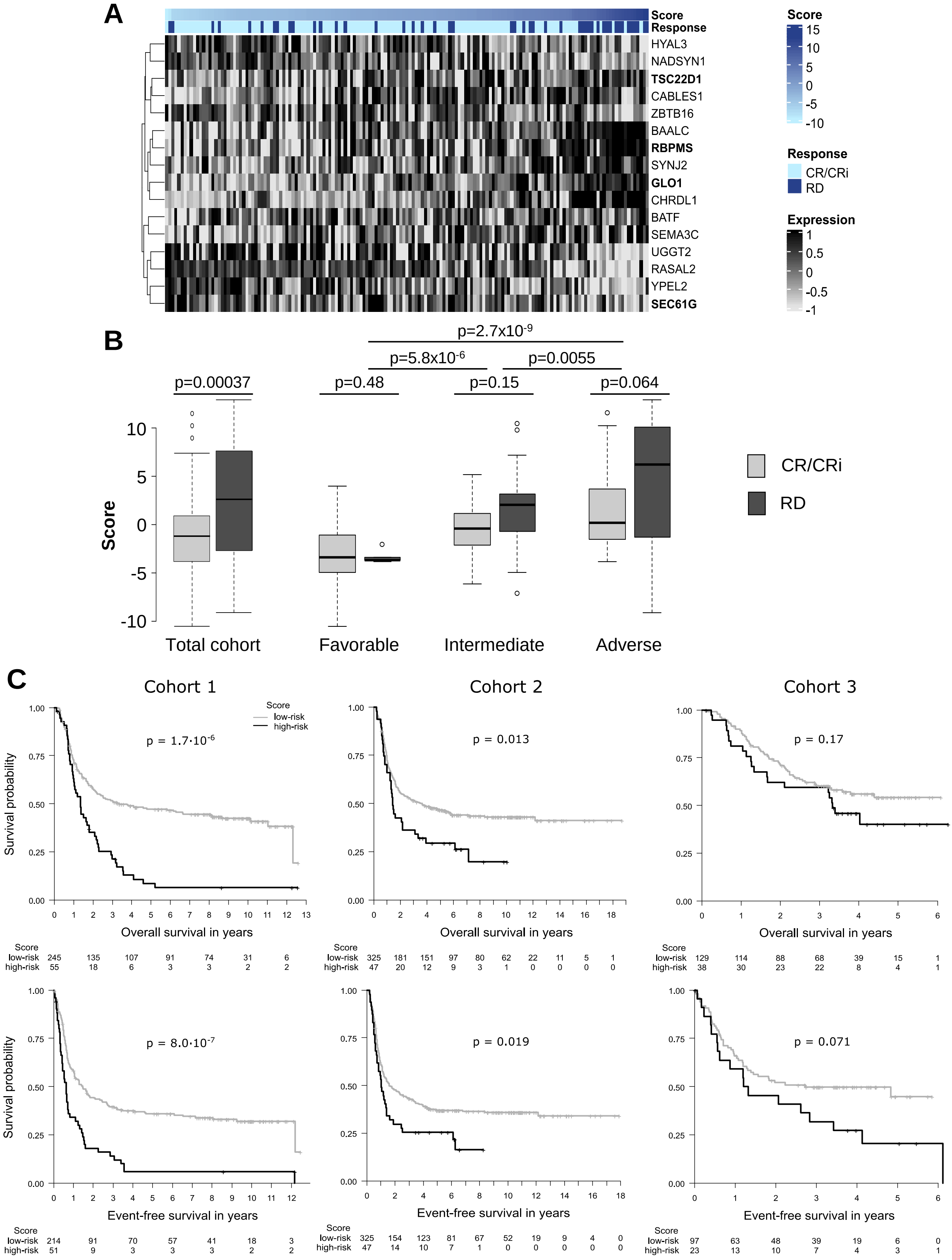
A
Score
Response
HYAL3
NADSYN1
TSC22D1
CABLES1
ZBTB16
BAALC
RBPMS
SYNJ2
GLO1
CHRDL1
BATF
SEMA3C
UGGT2
RASAL2
YPEL2
SEC61G
Score
15
10
5
0
-5
-10
Response
CR/CRi
RD
Expression
1
0.5
0
-0.5
-1
B
p=2.7x10^-9
p=5.8x10^-6
p=0.0055
p=0.00037
p=0.48
p=0.15
p=0.064
Score
10
5
0
-5
-10
Total cohort
Favorable
Intermediate
Adverse
CR/CRi
RD
C
Cohort 1
Cohort 2
Cohort 3
Survival probability
Score
low-risk
high-risk
p = 1.7·10^-6
p = 0.013
p = 0.17
Overall survival in years
Score
low-risk 245 135 107 91 74 31 6
high-risk 55 18 6 3 3 2 2
Score
low-risk 325 181 151 97 80 62 22 11 5 1
high-risk 47 20 12 9 3 1 0 0 0 0
Score
low-risk 129 114 88 68 39 15 1
high-risk 38 30 23 22 8 4 1
p = 8.0·10^-7
p = 0.019
p = 0.071
Event-free survival in years
Score
low-risk 214 91 70 57 41 18 3
high-risk 51 9 3 3 3 2 2
Score
low-risk 325 154 123 81 67 52 19 9 4 0
high-risk 47 14 10 7 1 0 0 0 0 0
Score
low-risk 97 63 48 39 19 6 0
high-risk 23 13 10 7 4 3 1

Figure S7. AML patients with a high score show poor event-free and overall survival, related to Figure 2H-J.

(A) Performance of the predictive score in the independent validation cohort BEAT. The columns of the heatmap were sorted by the predictive score (from low to high), and all genes were scaled to the mean of 0 and variance of 1. The heatmap shows an enrichment of patients with RD on the far right where the score values are the highest.

(B) Association between the predictive score and the risk groups as defined by the European Leukemia Net (ELN) classification 2017 in the validation cohort BEAT (157 patients; RD: n = 46, CR: n = 107, CRi: n = 4), and between CR/CRi and RD patients within the ELN risk groups. All p-values were calculated with a two sided t test. CR: complete remission; CRi: complete remission with incomplete count recovery; RD: refractory disease.

(C) Kaplan-Meier plots showing the association of the predictive score with overall survival and event free survival in patients who achieved complete remission (CR or CRi) after induction treatment. The continuous score was dichotomized so that the proportion of high-risk patients corresponds to the proportion of refractory patients in the total cohort. The plots are shown in the 3 patient cohorts used for the preselection of significant genes. Numbers below the x-axis show the patients at risk.
